# Disruption of vacuolar protein sorting components of the HOPS complex leads to enhanced secretion of recombinant proteins in *Pichia pastoris*

**DOI:** 10.1186/s12934-019-1155-4

**Published:** 2019-07-03

**Authors:** Lukas Marsalek, Verena Puxbaum, Markus Buchetics, Diethard Mattanovich, Brigitte Gasser

**Affiliations:** 1 Austrian Centre of Industrial Biotechnology (ACIB GmbH), Muthgasse 11, 1190 Vienna, Austria; 2 Department of Biotechnology, BOKU-University of Natural Resources and Life Sciences, Muthgasse 18, 1190 Vienna, Austria; 3 Present Address: BHAK/BHAS Stegersbach, Kirchengasse 44, 7551 Stegersbach, Austria

**Keywords:** *Komagataella*, Vacuolar protein sorting, Recombinant protein production, Yeast, Protein secretion, Secretion enhancing factor

## Abstract

**Background:**

The yeast *Pichia pastoris* is a widely used host for the secretion of heterologous proteins. Despite being an efficient producer, we observed previously that certain recombinant proteins were mistargeted to the vacuole on their route to secretion. Simultaneous disruption of one vacuolar sorting pathway together with vacuolar proteases prevented this mis-sorting and resulted in higher levels of secreted heterologous protein. Inspired by the positive results, we now set out to investigate the influence of further parts of the vacuolar pathway, namely the Cvt-pathway and the homotypic fusion and protein sorting (HOPS) complex.

**Results:**

Strains impaired in the Cvt pathway (∆*atg11*, ∆*atg8*) had no effect on secretion of the model protein carboxylesterase (CES), but resulted in lower secretion levels of the antibody fragment HyHEL-Fab. Disruption of genes involved in the HOPS complex led to vacuole-like compartments of the B category of *vps* mutants, which are characteristic for the deleted genes *YPT7, VPS41* and *VAM6*. In particular ∆*ypt7* and ∆*vam6* strains showed an improvement in secreting the model proteins HyHEL-Fab and CES. Additional disruption of the vacuolar protease Pep4 and the potential protease Vps70 led to even further enhanced secretion in ∆*ypt7* and ∆*vam6* strains. Nevertheless, intracellular product accumulation was still observed. Therefore, the secretory route was strengthened by overexpression of early or late secretory genes in the vacuolar sorting mutants. Thereby, overexpression of Sbh1, a subunit of the ER translocation pore, significantly increased HyHEL-Fab secretion, leading up to fourfold higher extracellular Fab levels in the ∆*ypt7* strain. The beneficial impact on protein secretion and the suitability of these strains for industrial applicability was confirmed in fed-batch cultivations.

**Conclusions:**

Disruption of genes involved in the HOPS complex, especially *YPT7*, has a great influence on the secretion of the two different model proteins HyHEL-Fab and CES. Therefore, disruption of HOPS genes shows a high potential to increase secretion of other recombinant proteins as well. Secretion of HyHEL-Fab was further enhanced when overexpressing secretion enhancing factors. As the positive effect was also present in fed-batch cultivations, these modifications likely have promising industrial relevance.

**Electronic supplementary material:**

The online version of this article (10.1186/s12934-019-1155-4) contains supplementary material, which is available to authorized users.

## Background

The yeast *Pichia pastoris* (syn. *Komagataella* spp.) has been extensively and successfully used to express heterologous secreted proteins [1–3]. In order for a protein to be secreted, it has to enter the lumen of the endoplasmic reticulum (ER) through the Sec61 translocon complex. In the ER lumen, the proteins become correctly folded by the help of chaperones such as Kar2 and other folding enzymes [4, 5]. The ER quality control machinery ensures that only correctly folded and modified proteins proceed on the secretory route to the Golgi apparatus. If some of the proteins fail to acquire a proper conformation, they are retained in the ER and destined for degradation by the ER associated degradation (ERAD) system [6, 7]. When the protein passes the quality control in the ER, it is allowed to be transported into the Golgi apparatus where it undergoes further modifications and awaits to be delivered to the cell membrane for secretion or to other cellular destinations.

Even though *P. pastoris* has been recognized as an efficient secretor with low levels of endogenously secreted proteins, the secretion capacity can be further increased by manipulating pathways within the cell that limit the overall secretion. Examples of successful cell engineering include the overexpression of folding helpers such as protein disulfide isomerase Pdi1, disruption of proteases, and overexpression of transcription factors such as the unfolded protein response (UPR) activator Hac1 or the oxidative stress response activator Yap1 (reviewed e.g. by [8, 9]). Recently, we showed that reducing vacuolar missorting by disruption of CORVET (“class C core vacuole/endosome tethering”) complex subunits led to enhanced secretion, and higher product titers could be achieved by combining CORVET mutants with the knock-out of vacuolar proteases [10]. Furthermore, we revealed that vacuolar degradation seems to be the prevalent degradative route in *P. pastoris*, and that ERAD mainly plays a role in degrading recombinant proteins failing to translocate and thus stuck at the cytosolic side of the translocon channel [11]. Based on these findings, we were intrigued if also other vacuolar sorting routes affect recombinant protein production.

The CPY and ALP pathways, named after their cargo proteins carboxypeptidase Y and alkaline phosphatase, respectively, are the main two pathways characterized to transport proteins from the late Golgi apparatus to the vacuole. While the CPY pathway represents an indirect pathway to the vacuole via the early endosome/multivesicular body (MVB), the ALP pathway mediates protein transport directly from the Golgi to the vacuole [12]. In order for the cargo to reach the vacuole, proper fusion of the interacting membranes has to take place. The fusion is mediated by Rab GTPase Ypt7 [13] and the two effector proteins Vam6/Vps39 and Vps41 [14] of the multisubunit tethering complex called “homotypic fusion and protein sorting” (HOPS) residing at the vacuolar membrane [15, 16]. Apart from these specific subunits, the HOPS complex is structurally related to the CORVET complex, and they are sharing four class C Vps protein subunits: Vps11, Vps16, Vps18, and Vps33. Through the activation of Rab GTPases by guanine nucleotide exchange factors (GEF), mediating the exchange of GDP for GTP, the tethers are able to capture and trap target vesicles prior to the membrane fusion mediated by *N*-ethylmaleimide-sensitive factor attachment protein receptors (SNAREs) [16].

Another way of vacuolar protein transport is autophagy or the closely related Cvt pathway (cytoplasm-to-vacuole targeting) where targeted proteins do not enter the ER but instead are transported directly from the cytoplasm to the vacuole [17]. Autophagosomes are created during macroautophagy as a response to stress such as nutrient limitation, whereas Cvt vesicles are formed under vegetative conditions to deliver resident hydrolases such as aminopeptidase I (Ape1) and α-mannosidase (Ams1) to the yeast vacuole. Depending on which pathway is activated, the target protein is recognized by either autophagosomes or Cvt vesicles, which fuse with the vacuole and release their cargo into the vacuolar lumen for degradation and subsequent recycling. In the filamentous fungus *Aspergillus oryzae*, mutants in autophagy-related genes (atg) led to enhanced secretion of bovine chymosin (Yoon et al. [18]). As Ypt7 has also been shown to be involved in macroautophagy and the Cvt pathway [19], we also included two proteins specifically involved in these pathways (Atg8, Atg11) in our study.

In order to characterize which routes might target recombinant proteins for degradation, we generated several *P. pastoris* strains impaired in either Cvt or the HOPS complex acting at the final steps of vacuolar protein sorting, and investigated their impact on secretion of two heterologous model proteins, the antibody fragment HyHEL-Fab [11] and the carboxylesterase from *Sphingopyxis* sp. MTA144, an enzyme that hydrolyzes antinutritive substances that may be naturally contained in animal feed [10, 20]. Both recombinant proteins were shown to be partly missorted to the vacuole in *P. pastoris* previously [10]. Furthermore, we have observed that translocation into the ER poses an additional bottleneck for the Fab fragment [11].

## Results

### Disruption of Atg8 and Atg11 involved in the macroautophagy/Cvt pathway does not affect heterologous protein secretion in *P. pastoris*

**Fig. 1:**
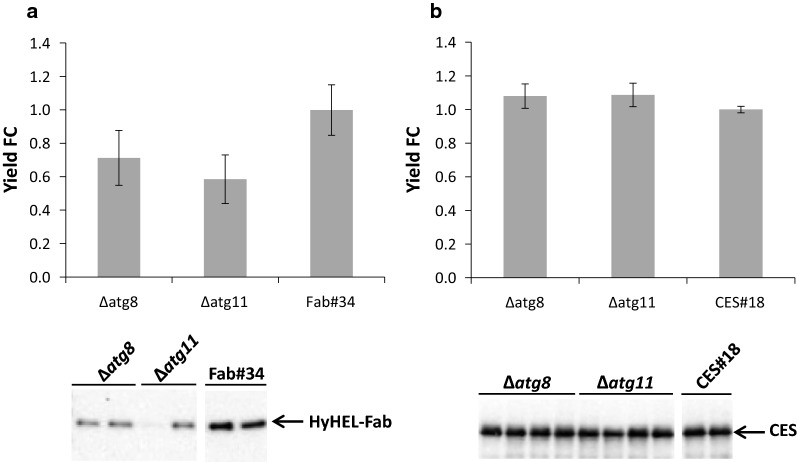
Effect of ∆*atg8 or* ∆*atg11* on model protein secretion in Fab#34 and CES#18. Detection of secreted HyHEL-Fab (**a**) and CES (**b**) by ELISA and Western blot. Prior to loading, the protein amounts were normalized to the wet cell weight of each strain. For ELISA, the relative fold changes of extracellular Fab yields were calculated from one screening with 2 ∆*atg8*, 2 ∆*atg11* clones and 2 Fab#34 biological replicates whereas the relative fold changes of extracellular CES yields were calculated from two screenings with 4 ∆*atg8*, 4 ∆*atg11* clones and 2 CES#18 biological replicates in each screening. Error bars indicate the standard error of the mean (SEM)

In order to prevent the undesired transport of the model proteins to the vacuole for degradation, we first focused on disrupting the autophagy-related Cvt pathway where selected proteins are packaged into Cvt vesicles in the cytoplasm and transported to the vacuole. Two genes (*ATG8*, *ATG11*) were chosen to be disrupted due to their important roles in biogenesis of pre-autophagosomal structure and cargo selection into Cvt vesicles, respectively [21–24]. The single gene disruptions were generated in the strain Fab#34 secreting the HyHEL-Fab antibody and the strain CES#18 secreting carboxylesterase. The secretion performance of the engineered strains was evaluated from the screenings and is shown in Fig. 1.

As can be seen from the Western blot and ELISA results, the disruption of *ATG8* and *ATG11* in both Fab#34 and CES#18 did not have a positive impact on secretion. In fact, the extracellular product yields were 40–50% lower (HyHEL-Fab secretors, Fig. 1a) or at similar levels (CES secretors, Fig. 1b) compared to the respective control strain. Biomass was not affected in full medium (CES secretors), while the ∆*atg8* and ∆*atg11* clones reached only 80% of the biomass concentration of the parent in minimal medium (Fab secretors). The lower secreted Fab levels in the two Cvt-engineered strains (Fig. 1a) are most probably due to the lower biomass accumulated during the screening (Additional file 1: Table S1), as there is no difference in intracellular Fab levels per biomass in these strains (Additional file 2: Figure S1). Our observations therefore reassure that the heterologous proteins found in the vacuole of *P. pastoris* [10, 25] were indeed missorted from the late secretory route, rather than being misfolded cytosolic forms. The Cvt pathway seems not to be involved in vacuolar targeting of heterologous secretory proteins, at least not in the analysed conditions.

### Disruption of HOPS complex subunits leads to enhanced protein secretion

Previously, we showed that recombinant CES was missorted to the vacuole and that disrupting the CORVET complex involved in the early steps of vacuolar sorting had a positive effect on recombinant protein secretion [10]. Encouraged by the positive results, we further investigated the vacuolar pathway, now focusing on the late vacuolar pathway and its impact on protein secretion efficiency. All vacuolar protein sorting pathways share the last steps of vesicle fusion with the vacuole, which is mediated by the Rab GTPase Ypt7 and its two effector proteins Vam6 and Vps41 of the HOPS complex [15].

**Fig. 2:**
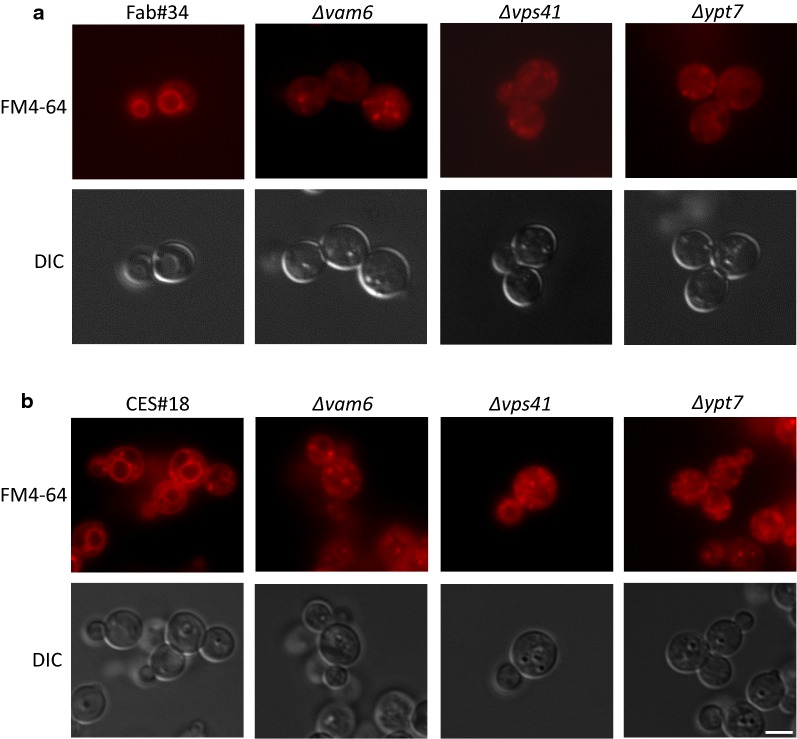
Vacuolar membrane staining of **a** Fab#34, ∆*vam6*, ∆*vps41,* ∆*ypt7* and **b** CES#18, ∆*vam6*, ∆*vps41*, ∆*ypt7*. The vacuolar membrane was stained with FM4-64 and the cells were viewed in an epifluorescence microscope. The fluorescence and the DIC images are shown. Per strain at least 300–500 cells were analysed in at least 10 microscopic images, of which 100% show the described phenotype. Two representative full size microscopic images per strain are shown in Additional file 2: Figure S2. Bar, 3 µm

To restrict vacuolar fusion and therefore potential product degradation of the two model proteins CES and HyHEL-Fab, several strains disrupted in Ypt7, Vam6 and Vps41 were generated in the background of the producing strains Fab#34 and CES#18. In order to check vacuolar morphology of these HOPS-engineered strains as well as to confirm the generation of positive transformants, fluorescence microscopy of FM4-64 stained cells was performed (Fig. 2). Upon disruption of genes involved in the HOPS complex, the cells did no longer contain one large vacuole as observed for the control strains, but instead, contained multiple small vacuole-like compartments. This phenotype of fragmented vacuoles is categorized in the B category of *vps* mutants as classified by Banta et al. [26] and Raymond et al. [27] and is characteristic, among others, for ∆*ypt7*, ∆*vam6* and ∆*vps41* strains in *S. cerevisiae* [28]. These small vacuole-like compartments are derived from the incapability of the vacuole to fuse with vesicles delivered through endocytosis or vacuolar targeting pathways, therefore allowing the intracellular vesicles to be accumulated in the cytoplasm. In each case, fluorescence microscopy confirmed the positive generation of ∆*ypt7*, ∆*vam6* and ∆*vps41* strains.

**Fig. 3:**
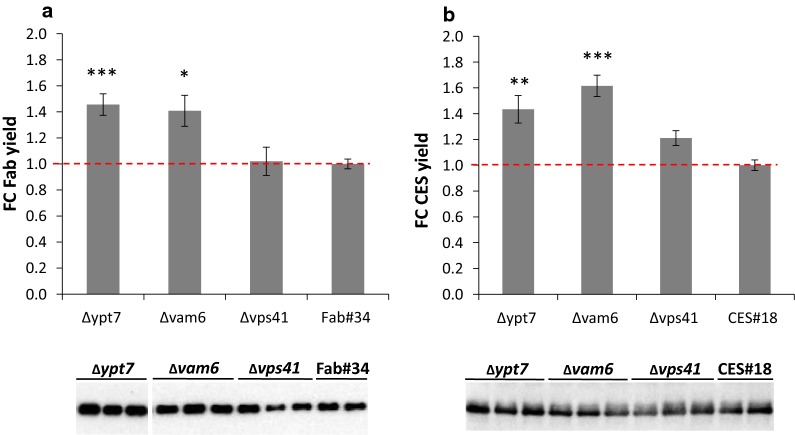
Screening of ∆*ypt7,* ∆*vam6* and ∆*vps41* strains overexpressing HyHEL-Fab or CES along with their respective control strains Fab#34 and CES#18. Detection of extracellular HyHEL-Fab (**a**) and CES (**b**) by ELISA and Western blot. For the ELISA, the relative fold changes of the yield were calculated from 3 to 5 biological and technical replicates each. Error bars represent the SEM. Statistical significance was measured by the Student’s *t* test and indicated with an asterisk (p < 0.05*, p < 0.01**). Prior to loading the samples on the Western blot, the protein amounts were normalized to the wet cell weight of each strain

After positive transformants were confirmed, HOPS mutant strains and their respective controls were cultivated in small scale screenings. After 48 h of incubation, Western blot was performed to evaluate the integrity of the product, and changes in secretion in each of the HOPS engineered strains were quantified by ELISA (Fig. 3). The Western blot confirmed intact product at the expected size (Fig. 3). The supernatants of HyHEL-Fab ∆*ypt7* and ∆*vam6* strains contained higher HyHEL-Fab concentrations than the control strain, outperforming it in yield by 46% and 41%, respectively (Fig. 3a). Similar observations were recorded also for CES secretors where the average yield of ∆*ypt7* and ∆*vam6* strains was higher compared to the control CES#18 by 40% and 73%, respectively (Fig. 3b). Even though Vps41 and Vam6 are both interacting with Ypt7 [29], the disruption of *VPS41* did not have the same effect on secretion as disruption of *VAM6* and *YPT7*. In fact, the secretion of both model proteins was at a similar level as for the controls supporting the findings of Harsay and Schekman [30] in *S. cerevisiae* that sorting of exocytic proteins is not affected in ∆*vps41* cells. Simultaneous disruption of two HOPS subunits ∆*vam6*∆*vps41* or ∆*vam6*∆*ypt7* had similar or even slightly lower improvement than single disruptions, indicating either metabolic burden or this effect is due to the fact that both subunits act in the same pathway (Additional file 1: Table S1). Since the secretion of both model proteins in the ∆*vps41* strain was not improved, further strain engineering was continued with the ∆*ypt7* and ∆*vam6* strains.

### Effect of disruption of the vacuolar associated proteins Pep4, Prb1 and Vps70 in Δ*ypt7* and Δ*vam6* strains

**Fig. 4:**
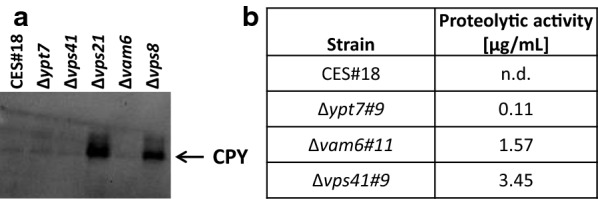
Extracellular carboxypeptidase Y and overall proteolytic activity present in the supernatant of HOPS and CORVET mutants. CPY in the supernatant was detected by Western blot (**a**) and the proteolytic activity was measured by a protease activity assay (**b**). Proteolytic activity is given as µg/mL tryptic equivalents. The technical variance of the method was approximately 10%. The results of one representative measurement are shown. nd: not detectable

**Fig. 5:**
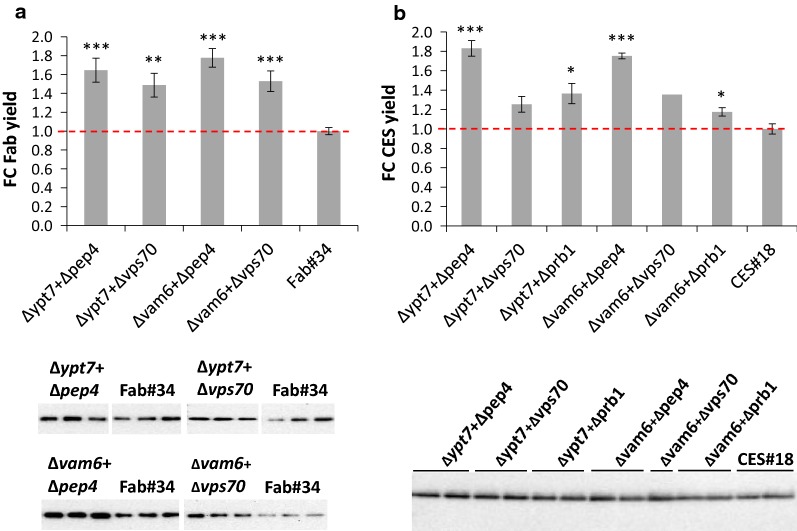
Screening of HOPS engineered strains with disrupted proteases. Detection of secreted HyHEL-Fab (**a**) or CES (**b**) by ELISA and Western blot. Prior to loading, the protein amounts were normalized to the wet cell weight of each strain. The relative fold changes of extracellular HyHEL-Fab yields (**a**) were calculated from 4 independent screenings with 3–6 clones of ∆*ypt7*∆*pep4*, ∆*ypt7*∆*vps70*, ∆*vam6*∆*pep4*, ∆*vam6*∆*vps70* and Fab#34 in each screening. For CES secretors (**b**), the relative fold change yields were calculated from one screening with 4–6 clones of ∆*ypt7*∆*pep4*, ∆*ypt7*∆*vps70*, ∆*ypt7*∆*prb1*, ∆*vam6*∆*prb1* and CES#18 (except ∆*vam6*∆*pep4* (2 biological replicates) and ∆*vam6*∆*vps70* (1 clone). Error bars indicate SEM. Statistical significance determined by the Student’s t-test is indicated with an asterisk (p < 0.05*, p < 0.01**, p < 0.001***)

So far the presented results indicate that ∆*ypt7* and ∆*vam6* strains secrete higher concentrations of both recombinant proteins. As cells with disrupted vacuolar protein sorting pathways have been reported to potentially over-secrete vacuolar proteases, we assessed the presence of vacuolar carboxypeptidase CPY in the supernatant of the engineered strains. In contrast to the high level of extracellular CPY found in the CORVET mutants (7–12 µg/mL tryptic equivalents; [10]), HOPS mutants did not strongly over-secrete vacuolar proteases (Fig. 4a). This is also reflected in the only slightly elevated proteolytic activities in the HOPS mutants compared to the parent strain (Fig. 4b). Nevertheless, we decided to investigate the impact of protease impairment in the HOPS-deficient mutants. Therefore, single gene disruptions of three vacuolar proteases were made in ∆*ypt7* and ∆*vam6* strains and the newly generated double-disrupted strains were screened for recombinant protein secretion (Fig. 5).

The three proteases chosen to be disrupted included Pep4, Prb1 and the potential protease Vps70 (PP7435_Chr1-1501). Pep4 (proteinase A) is an aspartyl protease involved in the posttranslational regulation of vacuolar hydrolases in *S. cerevisiae* [31], while Prb1 (proteinase B) is a serine protease of the subtilisin family and its activity depends on the levels and function of Pep4 [32, 33]. Both ∆*pep4* and ∆*prb1* have previously been associated with enhanced protein secretion in several host organisms including *P. pastoris* [34–36]. The function of the last protein, Vps70, is not fully known but it contains a protease-associated domain. Besides its potential function as a protease, this gene was also down-regulated in microarray analysis of HyHEL-Fab producing strains in chemostat cultivations (unpublished data).

Additional disruption of *PEP4* and *VPS70* in ∆*ypt7* and ∆*vam6* strains improved the secretion yield of HyHEL-Fab (Fig. 5a). Compared to the control strain Fab#34, ∆*ypt7*∆*pep4* and ∆*ypt7*∆*vps70* mutants outperformed the control in extracellular Fab yield by 51% and 62%, respectively. Even higher differences to the control Fab#34 were reported for ∆*vam6*∆*pep4* and ∆*vam6*∆*vps70* where the Fab yield was improved by 87% and 68%, respectively. The double disrupted strains ∆*ypt7*∆*pep4* and ∆*vam6*∆*pep4* reached 15–30% higher titers and yields compared to the single disrupted ∆*ypt7* and ∆*vam6* strains (Additional file 1: Table S1), with only ∆*vam6*∆*pep4* showing a statistically significant increase of 1.26-fold higher Fab yields in comparison to ∆*vam6*. However, this occurrence of increased secretion upon protease disruption was not observed for ∆*ypt7*∆*prb1* and ∆*vam6*∆*prb1* strains where significantly lower amounts of HyHEL-Fab than in the control Fab#34 were detected. As already seen previously [10], the disruption of Prb1 alone had a negative effect on HyHEL-Fab secretion, which was also observed in combination with ∆*ypt7* or ∆*vam6* in this study, indicating that Prb1 might be involved in other functions affecting secretion of this model protein rather than proteolysis. Indeed, fluorescence microscopy revealed an altered pattern of FM4-64 staining in ∆*ypt7*∆*prb1* that was not observed for any other protease knockout (Additional file 2: Figure S3).

Additional disruption of vacuolar proteases did not have the same impact on secretion in ∆*ypt7* and ∆*vam6* strains secreting CES (Fig. 5b). In fact, only the additional disruption of Pep4 in the ∆*ypt7* and ∆*vam6* strains resulted in higher yields as compared to single disrupted strains, outperforming the control strain CES#18 in yield by 83% and 75% for ∆*ypt7*∆*pep4* and ∆*vam6*∆*pep4*, respectively. Disruption of *VPS70* and *PRB1* did not result in such a high increase to the control but still reaching up to 36% yield improvement in ∆*ypt7*∆*prb1* followed by ∆*vam6*∆*vps70* (35%), ∆*ypt7*∆*vps70* (25%) and ∆*vam6*∆*prb1* (18%). However, when compared to the yield of single disrupted ∆*ypt7* and ∆*vam6* strains, the double disrupted strains did not show any improvement in secretion. This indicates that in HOPS mutants Pep4 is the main responsible protease for product degradation. In contrast, disruption of *PRB1* in HOPS engineered strains failed to enhance secretion significantly and even lowered the yield of single disrupted strains. These findings were especially surprising in the case of CES production where our previous results identified Prb1 to be the main responsible protease for CES degradation in CORVET engineered strains ∆*vps8* and ∆*vps21*, significantly enhancing secretion by 52% and 80%, respectively [10]. Thus, for further experiments either the single HOPS mutants or the combination with ∆*pep4* were used.

### Intracellular amounts of heterologous product in ∆*ypt7 and* ∆*vam6* strains

**Fig. 6:**
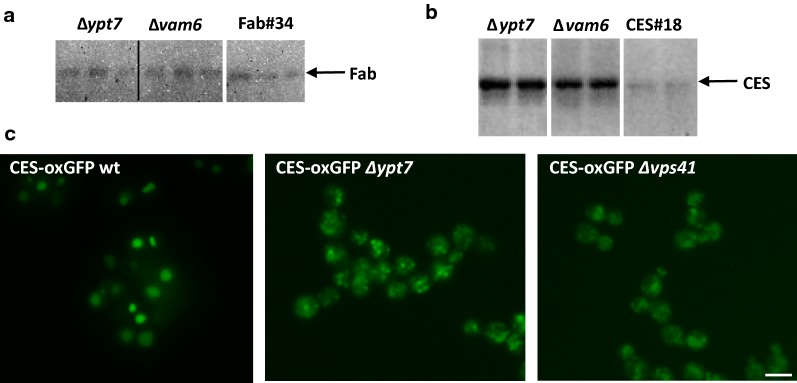
Intracellular accumulation of the recombinant protein in wt, Δ*ypt7* and Δ*vps41* strains. Intracellular levels of HyHEL-Fab (**a**) and CES (**b**) were detected by Western blot of cell lysates. Equal amounts of total intracellular protein (determined by BCA assay after cell lysis) were loaded on the gel. **c** Localization of the recombinant protein in *P. pastoris* overexpressing CES-oxGFP in wt, Δ*ypt7* and Δ*vps41* strains. After cultivation cells were put directly on a slide and viewed in an epifluorescence microscope. Bar, 5 µm

Next, we followed up what happens to the intracellular product in the ∆*ypt7* and ∆*vam6* strains where vacuole formation is impaired (Fig. 6a, b). Compared to the control strain CES#18, the two mutants accumulated higher amounts of CES intracellularly (Fig. 6b). In the Fab strains, higher intracellular product concentrations were only found in strains lacking also vacuolar proteases (not shown), but not in the single HOPS mutants (Fig. 6a), indicating that intracellularly accumulated/missorted Fab was readily degraded when vacuolar proteases were present, while CES is more stable to vacuolar degradation in the HOPS mutants. Fluorescence microscopy revealed that in ∆*ypt7* and ∆*vam6* the intracellular product (exemplified here as CES-oxGFP) was located to the punctuate structures (Fig. 6c) that are most likely representing the pre-vacuolar compartments characteristic for the HOPS mutant strains (as shown in Fig. 2). Intracellular product retention despite manipulating the vps pathway was also observed previously e.g. in *Schizosaccharomyces pombe,* where intracellular human growth hormone (hGH) still accumulated in protease deficient *Δvps10*, *Δvps22*, and *Δvps34* mutants [37].

As there was still intracellular non-secreted product observed in the HOPS mutants strains for CES and Fab (Fig. 6a, b), we next sought to completely block vacuolar transport by combining them with CORVET knockouts. Double disruption of CORVET and HOPS subunits (∆*vam6*∆*vps8*, ∆*vam6*∆*vps21*, ∆*ypt7*∆*vps8*, ∆*ypt7*∆*vps21*) or disruption of the shared subunits (Vps16, Vps33) was attempted. However, except for ∆*vam6*∆*vps8* in the CES#18 background, we were not able to generate any double mutants of the two pathways. It was not possible to combine disruptions of genes encoding both RabGTPases (Vps21 and Ypt7), or one Rab GTPase and a subunit of the other complex, pointing to the assumption that one of the vacuolar pathways is needed for the survival of the cells. Similarly, disruption of the class C subunits Vps16 or Vps33 that are present in both the HOPS and the CORVET complex resulted in the same lethal phenotype. These results are different to *S. cerevisiae*, where strains with individual knockouts of class C subunits are viable [38].

Regarding CES secretion, ∆*vam6*∆*vps8* behaved like ∆*vps8* with significantly reduced product titers (Additional file 1: Table S1). Upon simultaneous disruption of Prb1, titers of ∆*vam6*∆*vps8*∆*prb1* were rescued to the level of ∆*vps8*∆*prb1*, showing that the simultaneous disruption of the HOPS and the CORVET complex did not cause a synergetic effect on secretion.

### Overexpression of Sbh1 significantly increases secretion of HyHEL-Fab

Based on the finding that there are still considerable amounts of recombinant product found intracellularly (Fig. 6), as a next step we aimed to reinforce the secretory pathway. As it seems that the retained intracellular product is already fully processed, according to the correct size (Fig. 6), and trapped in the fragmented vacuolar vesicles (Fig. 6c), we decided to overexpress secretion enhancing factors in the HOPS mutants. As there was previous evidence that HyHEL-Fab is facing bottlenecks during folding and secretory transport [11, 39], we decided to focus on this model protein.

The genes to be overexpressed were selected based on previous microarray analysis, where tens of genes were identified to be significantly upregulated in HyHEL-Fab producing strains compared to a non-producing control cultivated in chemostat cultures [39]. Out of these, 10 genes were shown to be beneficial for Fab secretion when co-overexpressed in the methanol based AOX1 expression system [39]. So far, no results in the GAP-based expression system were available. To cover a wide range of the secretory pathway functions, 3 of these factors, Kar2, Sbh1 or Rho4, were chosen to be individually overexpressed in Fab#34 under the control of the P_GAP_ promoter.

Kar2 acts as a chaperone to mediate protein folding in the ER and regulates UPR via interaction with Ire1. Overexpression of this helper factor has already been proven to have a positive effect on heterologous protein secretion in some cases, while no impact or even negative effects were reported for other cases (reviewed e.g. by [40]). The second target gene *SBH1* encodes the beta subunit of the Sec61 ER translocation complex engaged in nascent peptide translocation into the ER [41]. In *S. cerevisiae* the beta subunit is encoded by two genes, *SBH1* and *SBH2*, and overexpression of the latter has been implicated with higher protein secretion [42]. The third gene selected for overexpression was PP7435_Chr3-0607, which most likely encodes the non-essential small GTPase Rho4 belonging to the Rho/Rac subfamily of Ras-like proteins. In *S. cerevisiae*, Rho4 was shown to be dispensable for cell growth, but it plays a role during cell separation where it regulates secretion of the hydrolytic enzymes required for cell septum degradation. Rho4 is also functionally related to Rho3. The interaction of both genes regulates bud formation and is involved in the establishment and maintenance of cell polarity [43, 44]. Interestingly, overexpression of *RHO4* in fission yeast results in a defective cell wall, suggesting an additional role for Rho4 in cell wall integrity [45].

**Fig. 7:**
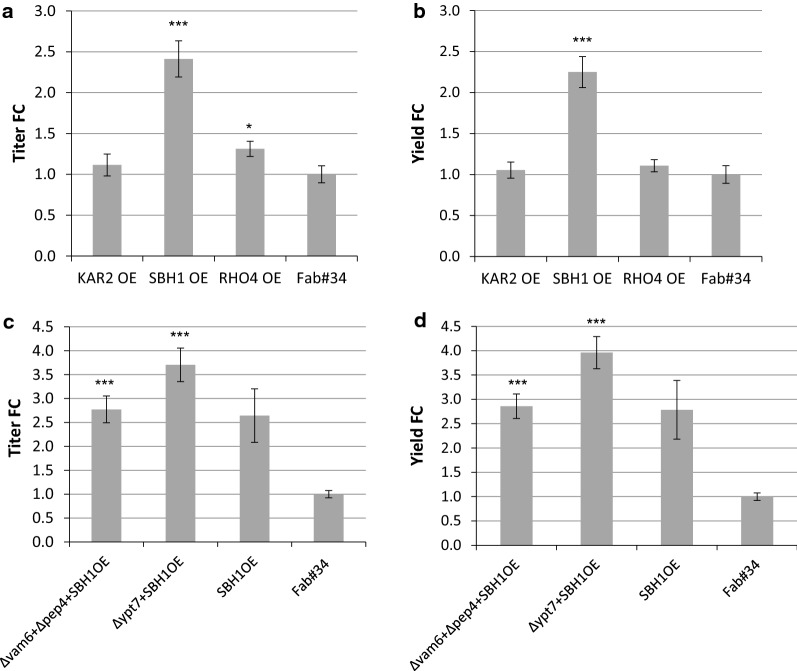
Screening of HyHEL-Fab#34 overexpressing *KAR2*, *SBH1* or *RHO4*, and Δ*vam6*Δ*pep4* or *Δypt7* overexpressing *SBH1*. The relative fold changes of extracellular HyHEL-Fab titers (**a**) and HyHEL-Fab yields (**b**) were determined by ELISA and calculated from one screening containing 12 clones of each strain including the empty vector control strain Fab#34. The relative fold changes of secreted HyHEL-Fab titers (**c**) and HyHEL-Fab yields (**d**) in the HOPS mutants were determined by ELISA and calculated from one screening containing the 4 best performing clones of Δ*vam6*Δ*pep4 *+ *SBH1*OE, Δ*ypt7 *+ *SBH1*OE, *SBH1*OE and Fab#34 strains. Error bars indicate the standard error of the mean (SEM). Statistical significance measured by the Student’s t-test is indicated with an asterisk (p < 0.05*, p < 0.01**, p < 0.001***)

Initially, each of the three genes or the empty vector control (EV) was overexpressed in Fab#34 and 12 clones of each construct were screened for Fab secretion. Kar2 did not cause any significant change in HyHEL-Fab secretion (Fig. 7a, b). In fact, titer and yield were decreased below the levels of the EV control strain by 7% in titer and 12% in yield, respectively. Similar results were also reported before [40, 46, 47] and explained by the fact that Kar2 accumulation could prevent activation of the UPR pathway leading to reduced activation of chaperone genes through Hac1 induction. The situation was different for the overexpression of *RHO4* where both measured values were higher compared to the control with an increase of 31% in titer and 11% in yield, respectively. Since Rho4 was reported to participate in the secretion of Eng1 and Agn1 glucanases, important for the septum degradation during cytokinesis [48], the higher extracellular concentrations of HyHEL-Fab could be a result of increased speed of cell division. This was also confirmed in our case where the average biomass of the *RHO4* overexpressing clones was increased by 10–20% compared to the EV control. The average titer of 12 clones overexpressing *SBH1* was more than twofold higher than the average titer of the empty vector control clones. Similarly, the yield amounted to nearly twofold increase compared to the EV control.

Since overexpressing *KAR2* did not yield any promising results in terms of higher secretion levels, only *SBH1* or *RHO4* were overexpressed in the Fab producer strains with disrupted HOPS components (Fig. 7c, d). Unexpectedly, the combination of *RHO4* overexpression with ∆*vam6* seems to cause a synthetically lethal phenotype, as no viable colonies could be obtained (also when trying a different order of cell engineering steps), which might indicate that they are functioning in closely related pathways. In the background of ∆*ypt7*, *RHO4* overexpression turned out to be successful, leading to 57% higher Fab yield. However, the Δ*ypt7 *+ *RHO4*OE strain had approximately 20% lower biomass at the end of the screenings compared to the EV control.

Again, overexpression of *SBH1* in the background of high Fab producers deficient in vacuolar transport, namely Δ*ypt7* and Δ*vam6pep4* strains, significantly outreached the secretion capacity of the control strain Fab#34 transformed only with the empty vector (Fig. 7c, d). *SBH1* overexpression—alone or in combination—led to a significant increase in HyHEL-Fab secretion. In the case of the Δ*ypt7 *+ *SBH1*OE strain, the clones again significantly outreached the secretion capacity of the control strain Fab#34 + EV with nearly fourfold higher titers and yields, respectively (Fig. 7c, d) while reaching similar biomass. With this high secretion performance, the Δ*ypt7 *+ *SBH1*OE strain exceeded the improvements of *SBH1* overexpression alone by 1.4-fold and ∆*ypt7* alone by more than 2.5-fold. Only a slightly beneficial effect was seen when overexpressing *SBH1* in the Δ*vam6*Δ*pep4* background compared to *SBH1* overexpression alone, again confirming that disruption of the RabGTPase had a higher impact than disruption of the other HOPS complex components. We conclude a synergistic effect of Sbh1 and Ypt7 due to an efficient transport of the product into the ER and less targeting to the vacuole.

### Fed-batch cultivations of HOPS engineered strains secreting HyHEL-Fab

To verify the performance of the HOPS engineered strains in a production process, fed-batch cultivations of the most promising engineered strains expressing HyHEL-Fab along with their parental strain were conducted. Standard glucose-limited fed batch experiments (constant feed of 3.4 g/h of the glucose fed-batch solution for 75 h fed-batch in 4-parallel DASGIP bioreactors) were performed (4 clones per run). For ∆*ypt7*, ∆*ypt7 *+ *SBH1OE* and ∆*vam6*∆*pep4 *+ *SBH1*OE duplicate fed batch runs were performed, while Fab#34 was cultivated in quadruplicates. The deviation between the replicate bioreactor runs of the Fab#34, Δ*vam6*Δ*pep4 *+ *SBH1*OE, Δ*ypt7* and Δ*ypt7 *+ *SBH1*OE was less than maximum 15% for Q_*P*_ and q_P_, and less than 10% for the biomass yield, indicating high consistency between the individual runs (Table 1 and Additional file 1: Table S2).

**Table 1:** Volumetric and specific productivity as well as product yield and specific growth rate of the fed-batch cultivation of HyHEL-Fab#34 overexpressing *SBH1*, deleted in *ypt7 and vam6pep4* and combinations thereof

	Mean Q_p_ [µg L^−1^ h^−1^]	Mean q_p_ [µg g^−1^ h^−1^]	Y_X/S_ [g g^−1^]	Mean µ [h^−1^]
Fab#34	0.25 ± 0.03	3.35 ± 0.28	0.443 ± 0.038	0.022 ± 0.001
Δ*vam6*Δ*pep4*	0.42	5.83	0.471	0.021
Δ*vam6*Δ*pep4 *+ *SBH1*OE	0.50 ± 0.06	6.36 ± 0.94	0.462 ± 0.012	0.0255 ± 0.0025
Δ*ypt*7	0.40 ± 0.03	5.85 ± 0.43	0.430 ± 0.043	0.023 ± 0.001
*SBH1*OE	0.42	4.81	0.367	0.021
Δ*ypt7 *+ *SBH1*OE	0.62 ± 0.00	9.36 ± 0.32	0.468 ± 0.008	0.0225 ± 0.0015

For Fab#34 mean ± SD from three individual fed batch cultivations are shown, for Δ*ypt7*, Δ*vam6*Δ*pep4 *+ *SBH1*OE and Δ*ypt7 *+ *SBH1*OE two individual fed batch cultivations were performed

**Fig. 8:**
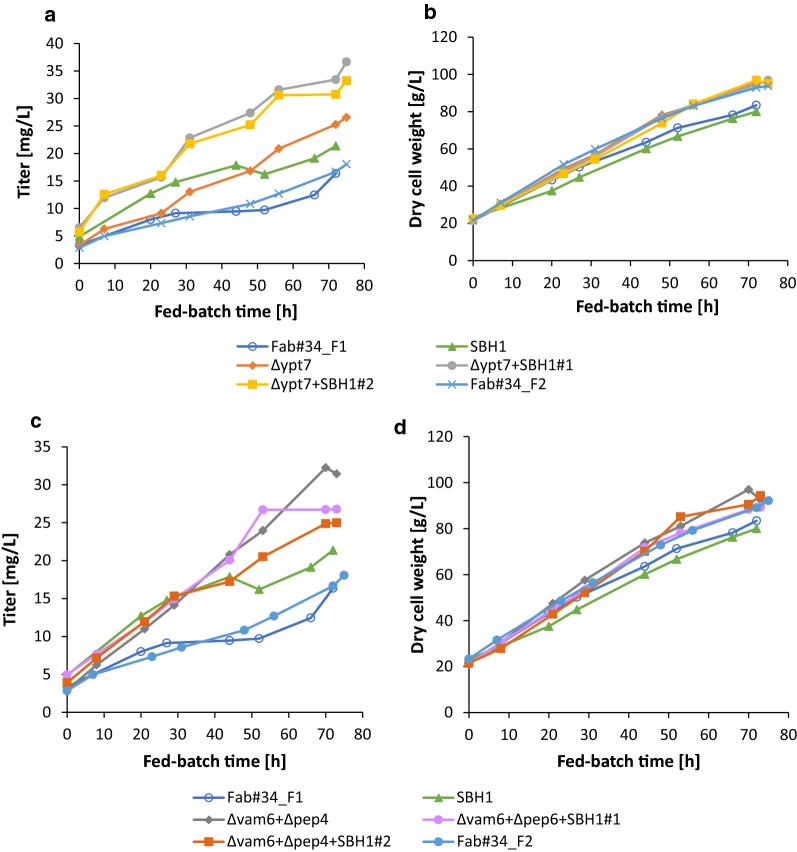
Fed-batch cultivation of HyHEL-Fab#34 overexpressing *SBH1* and deleted for *ypt7* or *vam6pep4* and combinations thereof. The product titers (**a**, **c**) of all the strains were determined by ELISA and measured at several time points during the fed-batch process. The dry cell weight (**b**, **d**) was assessed at similar time points of the cultivation

The batch phase was finished when the glycerol in the batch medium was consumed (after 24 ± 2.5 h). Biomass concentration was 21.9 ± 0.6 g/L DCW at batch end for all strains. During the course of the fed-batch cultivations, all the engineered strains secreted higher amounts of HyHEL-Fab than the control strain Fab#34, while reaching almost similar biomass concentrations (Fig. 8). The increase in extracellular Fab was also reflected in other characteristics such as average volumetric productivity and average specific productivity which turned out to be higher for the engineered strains (Table 1).

Interestingly, the ∆*vam6*∆*pep4 *+ *SBH1*OE strain outperformed the ∆*vam6*∆*pep4* strain only in initial stages of the fed-batch process, whereas towards the end the extracellular product concentration equaled or was even lower compared to the ∆*vam6*∆*pep4* strain (Fig. 8c). Nevertheless, over the whole process time the average specific productivity ∆*vam6*∆*pep4 *+ *SBH1*OE was increased by 1.9-fold over the control Fab#34, and 30% compared to single *SBH1* overexpression.

In the ∆*ypt7* background, the positive impact of *SBH1* overexpression on Fab secretion could be clearly observed and the secretion behavior of ∆*ypt7 *+ *SBH1OE* was easily distinguished from the performance of individual single mutations (Fig. 8a, b). Over the whole process time, the ∆*ypt7 *+ *SBH1*OE strains on average had a 2.8-fold higher specific productivity and a 2.5-fold higher volumetric productivity/space time yield than the control strain Fab#34 (Table 1). The final product titer was 1.9-fold increased (Fig. 8a). The double engineered strain ∆*ypt7 *+ *SBH1*OE outperformed the respective single engineered strains by 1.6- and 1.9-fold for ∆*ypt7* and *SBH1*, respectively. The results of these experiments correlated well with the trend observed in the screenings (Fig. 7), showing nicely the positive impact of disrupting a gene involved in vacuolar sorting as well as combining this deficiency with a secretion enhancing “helper factor” to enhance secretion even more.

## Discussion

Within the scope of this work, we have generated several *P. pastoris* mutant strains engineered in different parts of the secretory pathway including the autophagy related Cvt pathway, the HOPS complex involved in the vacuolar sorting pathways, as well as vacuolar proteases and factors involved in protein secretion. Various gene combinations were characterized and evaluated based on their secretion efficiency of two heterologous proteins, the antibody fragment HyHEL-Fab and carboxylesterase CES, in order to generate highly efficient production strains capable of exceeding the capacity of the control strains Fab#34 and CES#18 in terms of recombinant protein secretion.

The first degradative pathway to be disrupted was the autophagy related Cvt pathway involved in transporting targeted proteins such as Ape1 and Ams1 from the cytoplasm to the vacuole. *ATG8* and *ATG11* were chosen to be disrupted due to their important roles in biogenesis of Cvt vesicles and cargo selection, respectively. However, the ∆*atg* mutants did not demonstrate any improvement regardless of the secreted product. In fact, the ∆*atg* mutants secreting HyHEL-Fab performed less efficiently as the control strain whereas similar secretion capabilities were observed for ∆*atg* mutants secreting CES. While our findings do not correlate with the previous studies in filamentous fungi where atg mutants outperformed the reference strains in extracellular levels of heterologous proteins (Yoon et al. 2013), they clarify that autophagy is not involved in degradation of secretory proteins in *P. pastoris*.

Since disruption of the Cvt pathway did not lead to secretion increase, we next followed up on targeting the last step of vacuolar protein sorting, which is the fusion of vesicles to the vacuole. For that purpose, three genes (*YPT7*, *VAM6*, *VPS41*) of the HOPS complex were chosen to be disrupted based on their function in vacuolar fusion events. Of these, ∆*vam6* and ∆*ypt7* had a clearly positive effect on the secretion of the two different recombinant proteins. In all the cases, combination of more than one gene involved in the HOPS complex was counterproductive and did not cause a synergetic effect on secretion. Double disruption of CORVET and HOPS subunits (∆*vam6*∆*vps8*, ∆*vam6*∆*vps21*, ∆*ypt7*∆*vps8*, ∆*ypt7*∆*vps21*) or disruption of the shared subunits (Vps16, Vps33) was attempted, but except for ∆*vam6*∆*vps8* no viable clones could be obtained in contrast to *S. cerevisiae*, again indicating that despite having the same players different regulatory mechanisms exist in the vacuolar protein sorting pathways of different yeasts. In particular, disruptions of the genes encoding both Rab GTPases (Vps21, Ypt7) proved to be lethal in both production strains, suggesting that at least one Rab GTPase is required for the survival of the cell. Furthermore, the three HOPS mutant strains (∆*vam6*, ∆*vps41* and ∆*ypt7*) also differ in some of their phenotypes between the two yeasts. While they all displayed the typical class B-specific “fragmented vacuole” phenotype, the *P. pastoris* HOPS mutants do not oversecrete vacuolar proteases and CPY secretion is as in the control strain in contrast to *S. cerevisiae* [28, 38]. The underlying reasons for this are not known, but might be correlated to the fact that the vacuolar cargo accumulates in the pre-vacuolar vesicles in these strains.

Proteins of the HOPS complex indeed proved to play an highly important role in secretion of recombinant proteins, as demonstrated for CES and HyHEL-Fab. Disruption of *YPT7* and *VAM6* increased secretion, both in small-scale screening format as well as in fed-batch cultivations. Interestingly, in our studies in *P. pastoris* higher product titers of CES and HyHEL-Fab were obtained in the HOPS mutants than in the CORVET mutants, wherease in the screens for enhanced secretion of cellulases performed in *S. cerevisiae* the CORVET mutants always had the better performance [49, 50]. This could be related to an enhanced proteolytic susceptibility of our two model proteins compared to the cellulolytic enzymes. Indeed, further impairment of vacuolar proteases [Pep4, Prb1 and Vps70 (PP7435_Chr1-1501)] in the HOPS mutants enhanced the achieved product titers even further, with ∆*pep4* having the most distinct impact.

As was shown in previous studies, the intracellular concentration of heterologous proteins is kept at low levels in yeast where functional proteases are present. This was also found to be true for our control strains Fab#34 and CES#18. However, upon disruption of *YPT7* and *VAM6*, the intracellular amounts either stayed at the same level for HyHEL-Fab secretors or were even increased for CES secretors. Our results therefore show that vacuolar product degradation is diminished in ∆*vam6* and ∆*ypt7* strains where vesicles cannot properly fuse with the vacuole anymore. Nevertheless, the mutations in the HOPS complex are leading to a certain intracellular product retention and accumulation in putative pre-vacuolar compartments. Thus, to rescue the intracellularly trapped recombinant protein, we enforced the flux towards the secretory pathway, by overexpressing one of three *P. pastoris* genes involved either in the early secretory pathway (ER import protein Sbh1, ER protein folding chaperone Kar2) and the late secretory trafficking steps (RhoGTPase Rho4). Both *SBH1* and *RHO4* overexpression alone or in combination with ∆*ypt7* enhanced recombinant protein secretion. The effect was not as pronounced for the overexpression of *SBH1* in ∆*vam6*, and strikingly enhanced levels of Rho4 proved to be synthetically lethal in ∆*vam6*. Altough Vam6 has previously been implicated to be the GEF of Ypt7 [51], it has later been clarified that Vam6 is involved in localization and recruitment of Ypt7 to the vacuolar membrane, and serves as interaction partner for the Mon1-Ccz1 GEF complex [52]. Rho4 is a GTPase involved in late vesicular trafficking and cell polarization [43, 53]. We might speculate that upon overexpression in ∆*vam6*, Rho4 starts to interact with the Mon1-Ccz1 complex, leading to its hyperactivation and thus cell lysis. Regarding the positive effect of *SBH1* overexpression, one possible explanation would be that overexpression of this component of the translocation pore might overcome the translocation defect observed for HyHEL-Fab previously [11]. *SBH1* (also called *SEB1*) was initially found as a multicopy suppressor of translocation defective strains in *S. cerevisiae* [54, 55], indicating that higher levels of this single factor can strengthen the translocation efficiency. However, Sbh1 has also been found to be physically connected with several complexes and processes involved in protein secretion but not directly linked to translocation such as the exocyst complex [56], the OST complex [57], or the reticulons [58]. Indeed, Toikkanen et al. [42] hypothesized that the interaction of Sbh1 with the exocyst is the major factor leading to improved secretion of native and recombinant proteins in *S. cerevisiae* [42], which might be another reason for the improved secretion of Fab upon *SBH1* overexpression observed also in our study.

## Conclusions

Our study confirmed that vacuolar mis-sorting of recombinant proteins in *P. pastoris* happens via the Golgi-to-vacuole sorting pathways and thus most likely affects correctly folded active proteins. Disruption of these pathways alone or in combination with vacuolar proteases leads up to 80% higher extracellular product titers of both tested model proteins, HyHEL-Fab and CES. Upon combining the HOPS mutants with overexpression of secretion enhancing factors, synergistic effects and up to nearly fourfold higher Fab secretion in the strain ∆*ypt7 *+ *SBH1*OE was achieved in screening cultures. These results could be verified in standard glucose-limited fed batch cultivations, where specific Fab productivity was enhanced 2.8-fold in ∆*ypt7 *+ *SBH1* compared to the control strain. Our results thus present a versatile method to enhance recombinant protein secretion by combining mutants in vacuolar protein sorting not only with protease knockouts, but also with enhanced secretion promoting reactions.

## Methods

### Strains and plasmids

The generation of the strain CES#18 expressing carboxylesterase (CES) and the strain Fab#34 expressing HyHEL-Fab were described before [10, 11, 59]. Briefly, the recombinant proteins were expressed under control of the *P. pastoris* GAP promoter, with the *S. cerevisiae* α-MF leader sequence for secretion and a Zeocin resistance marker cassette. Prior to transformation, the expression vectors based on plasmid pPM2dZ30-PGAPα, a derivative of pPUZZLE [60], were linearized with the restriction enzyme *Avr*II for homologous integration into the native GAP promoter locus of the genome of the *Komagataella phaffii* wild type strain CBS7435 (Centraalbureau voor Schimmelcultures, NL). The three secretion factors *KAR2*, *RHO4* and *SBH1* were amplified from genomic DNA and overexpressed under control of the GAP promoter in the plasmid pPM2aK30, which contains the KanMX resistance marker cassette and the 3′-AOX1 region for genomic integration. The vector was linearized with *Asc*I prior to transformation.

### Disruption of genes involved in vacuolar transport and vacuolar proteases

The split marker cassette approach as described by Heiss et al. [61] was used to disrupt the genes. Therefore, the flanking regions of the split marker cassette used for homologous recombination (A upstream, D downstream) were first amplified by PCR along with the G418/Hygro resistance cassette fragments B and C. After each of the four fragments was amplified and purified, another round of PCR was performed to fuse A + B and C + D fragments together. After gel purification, equal amounts of both split marker fragments (AB and CD) were pooled and simultaneously transformed into electro-competent *P. pastoris*. Transformation was done by electroporation as described in [62]. The transformed cells were then plated onto selective YPD plates containing 50 μg/mL Zeocin + 500 μg/mL G418 or 200 μg/mL Hygromycin for single gene disrupted strains and 50 μg/mL Zeocin + 500 μg/mL G418 + 200 μg/mL Hygromycin for multiple gene disrupted strains. Positive transformants were verified by PCR of the genomic DNA using a detection primer pair designed to bind outside of the split-marker cassette in the *P. pastoris* genome (Additional file 2: Figure S4). Prior to confirming the positive transformants, the genomic DNA was isolated by the DNeasy Blood & Tissue Kit (Qiagen). All the primers used to disrupt the corresponding genes and detect positive transformants are summarized in Additional file 1: Table S3.

### Media and cultivation

Chemicals for media preparation were purchased from BD, Carl Roth, and Merck. YPD medium contained per liter 20 g peptone, 10 g yeast extract and 20 g glucose whereas YPD-agar additionally contained 20 g agar–agar. The minimal M2 medium used for the main culture for HyHEL-Fab secreting strains in screenings contained per liter: 3.15 g (NH_4_)_2_HPO_4_, 0.49 g MgSO_4_*7H_2_O, 0.80 g KCl, 0.0268 g CaCl_2_*2H_2_O, 22.0 g citric acid monohydrate, 1470 µL trace salts and 2 mL biotin (0.2 g/L). The pH of the M2 minimal medium was set to pH = 5.

The PTM0 trace salts stock solution contained (per liter) 6.0 g CuSO_4_·5H_2_O, 0.08 g NaI, 3.0 g MnSO_4_·H_2_O, 0.2 g Na_2_MoO_4_·2H_2_O, 0.02 g H_3_BO_3_, 0.5 g CoCl_2_, 20.0 g ZnCl_2_, 65.0 g FeSO_4_·7H_2_O and 5.0 ml H_2_SO_4_ (95 to 98%). All chemicals for PTM0 trace salts stock solution were from Riedel-de Haën (Seelze, Germany), except for H_2_SO_4_ (Merck Eurolab).

The buffered BM-medium, used for screenings of CES secreting strains as a main culture medium, contained per liter: 10 g yeast extract, 20 g soy peptone, 100 mM potassium phosphate buffer (pH 6.0), 13.4 g yeast nitrogen base without amino acids and 1 mL biotin stock solution (0.2 g/L).

The strains were cultivated in 24 deep well plates as described before [10, 11]. For the preculture, 2 mL of selective YPD medium was used containing different types of antibiotics according to the type of strain applied. In general, the control strains carrying only HyHEL-Fab or CES were grown in the presence of 50 μg/mL Zeocin, while single and multiple gene disrupted strains were grown in YPD medium supplemented with 50 μg/mL Zeocin + 500 μg/mL G418 and 50 μg/mL Zeocin + 500 μg/mL G418 + 200 μg/mL Hygromycin, respectively. The respective strains were inoculated using a sterile pipette tip and incubated overnight at 25 °C at 280 rpm. The cells were harvested and washed with M2/BM medium. The OD_600_ of the washed cells was measured and the main culture containing 2 mL M2 minimal medium/BM complex medium supplemented with one glucose feed bead (12 mm, Kuhner, CH) was inoculated with the starting OD_600_ = 1 (M2 medium) and OD_600_ = 0.1 (BM medium), respectively. The main culture was incubated for 48 h at 25 °C at 280 rpm. Feed beads are a polymer-based slow release system for controlled glucose release in shake flask and deep well plate cultivations, which allow for a glucose-limited cell growth in fed batch mode [63]. Growth kinetics are shown in Additional file 2: Figure S5. The cells were then harvested by centrifugation for 5 min at full speed and the supernatant was collected.

### Fed-batch cultivation

For the preculture, 200 mL of selective YPD medium containing antibiotics according to the type of strain applied were inoculated with one cryo tube of the working cell bank. After 24 h of incubation, the preculture was washed in the batch medium and used to inoculate 450 mL batch medium to reach an initial OD_600_ = 1.0. Fed-batch cultivations were carried out in 1.0-L working volume DASGIP bioreactors (Eppendorf, Germany) with a computer-based process control. The temperature was maintained at 25 °C, pH was controlled at 5.0 with 25% ammonia and the dissolved-oxygen concentration was kept above 20% saturation by controlling the stirrer speed and the airflow.

The batch medium contained (per liter) 2.0 g citric acid monohydrate, 12.6 g (NH_4_)_2_HPO_4_, 0.022 g CaCl_2_·2H_2_O, 0.9 g KCl, 0.5 g MgSO_4_·7H_2_O, 46.5 g glycerol, 4.6 mL PTM0 trace salts stock solution and 2 mL Biotin (0.2 g/L). The pH was adjusted to 5.0 with 25% HCl.

The glucose fed-batch solution contained (per liter) 464 g glucose·H_2_O, 8.4 g KCl, 5.2 g MgSO_4_·7H_2_O, 0.28 g CaCl_2_·2H_2_O, and 10.1 mL PTM0 trace salts stock solution and 1.70 mL Biotin (0.2 g/L).

The batch phase was finished when the glycerol in the batch medium was consumed (after 24 ± 2 h). Biomass concentration was 21.9 ± 0.6 g/L DCW at batch end for all strains. Afterwards, the glucose fed batch with a constant feed rate of 3.4 g /h 50% (w/v) glucose was started. The fed-batch phase was terminated at approximately 75 h. Samples were taken frequently and harvested by washing the pellets and centrifugation for 5 min at full speed. The supernatant was then collected and kept frozen at − 20 °C.

### SDS-PAGE and Western blot

The SDS-PAGE was performed using sodium dodecyl sulfate (SDS) NuPAGE^®^ 12% Bis–Tris polyacrylamide gels (Life Technologies™) with NuPAGE^®^ morpholinepropanesulfonic acid (MOPS) buffer at 180 V for 60 min. Before loading the gel with 15 μL of culture supernatant, each sample supernatant was correlated to the wet cell weight of each sample using RO water.

After SDS-PAGE, the separated proteins were transferred to a nitrocellulose membrane using the XCell II™ Blot Module for wet (tank) transfer (Life technologies™) according to the manufacturer’s instructions. The HyHEL-Fab was immunologically detected with Anti-Human IgG antibody (Abcam, ab7497) produced in mouse, specifically recognizing the Hinge region of Human IgG and Anti-Mouse IgG antibody (Sigma-Aldrich, A3673) coupled with HRP, which was produced in goat. Carboxylesterase was immunologically detected with rabbit anti-CES antiserum (produced in rabbit, provided by Biomin Holding GmbH) and carboxypeptidase Y was detected using anti-CPY antiserum (produced in rabbit, kindly provided by Günther Daum/Karlheinz Grillitsch, Graz University of Technology). As secondary antibodies, anti-rabbit IgG antibodies coupled with horseradish peroxidase (produced in goat, Sigma-Aldrich, A0545) were used.

### Quantification of HyHEL-Fab and CES by ELISA

Quantification of HyHEL-Fab by ELISA was done using anti-Human IgG antibodies (Sigma-Aldrich, A8542) coupled with alkaline phosphatase, produced in goat. The supernatant samples were serially diluted on precoated immunosorbent plates (Maxisorp; Nunc, Denmark) along with the purified HyHEL-Fab (Bethyl P80-115) used as standard with an initial concentration of 100 ng/mL. Detection was done with pNPP (Sigma-Aldrich S0942) dissolved in 0.1 M NaHCO_3_ buffer, pH 9.6–9.8. After each incubation step the plates were washed three times using washing buffer (PBS containing 0.1% Tween 20 adjusted to pH 7.4). For plate coating, standard PBS, pH = 7.4 was used whereas dilution steps were done in dilution buffer based on washing buffer additionally supplemented with 1% BSA (w/v).

The same procedure was applied to quantify CES by ELISA using the following antibodies: rabbit-anti-CES antiserum 6287 (produced and provided by Biomin Holding GmbH) as a coating antibody, guinea pig-anti-CES antiserum 12206 (produced and provided by Biomin Holding GmbH) as a primary antibody and goat-anti-guinea pig-IgG-alkaline phosphatase conjugate (Sigma-Aldrich, A-5062) as a detection antibody. The purified standards were produced by Biomin Holding GmbH and diluted to the initial concentration of 100 ng/mL. The yellow color reaction was developed using pNPP dissolved in the detection buffer as previously described. For plate coating, 100 mM carbonate buffer (pH = 9.6) was used.

The yield of recombinant proteins was calculated by relating the product titer to the wet cell weight concentration of the respective culture.

### Protease activity assay

Protease activity was measured by Pierce Protease Assay Kit according to the manufacturer’s instructions with slight modifications. The culture supernatants containing complex BM medium were washed and buffer exchanged using 2 mL Vivaspin columns (Sartorius) as instructed in the manual. Since we were interested in vacuolar proteases active at cultivation pH, the supernatants were reconstituted in 100 mM potassium phosphate buffer, pH = 6. The digestion steps as well as the TNBSA (trinitrobenzenesulfonic acid) development step were incubated for 1 h and overnight, respectively. The buffer exchange was not necessary for samples incubated in M2 medium. However, the incubation times stayed the same.

### Fluorescence microscopy

HyHEL-Fab and CES expressing *P. pastoris* was inoculated in M2/BM medium with an initial OD_600_ of 0.1 and grown for approximately 18 h. Vacuolar membranes of the cells were stained using FM4-64 [64] as described previously [65]. Briefly, the cells were stained with 15 µM FM4-64 (Invitrogen) diluted in culture medium for 15 min at 30 °C shaking in the dark. The cells were washed and incubated for 1 h in culture medium. After a final washing step, the cells were viewed on a Leica DMI6000B fluorescence microscope using a HCX PL APO CS 100.0 × 1.40 NA oil-immersion objective and appropriate filters for FM4-64 (Leica N2.1). For CES-oxGFP, cells were cultivated in BM medium to an OD of approx. 1.5–2. The cells were then centrifuged (700*g*, 3 min), resuspended in 1× PBS and viewed in the microscope using a filter for GFP (Leica L5). Images were processed using ImageJ (Rasband W.S. ImageJ, U.S. National Institutes of Health, Bethesda, MD, USA, http://imagej.nih.gov/ij/, 1997–2018).

## Additional files

**Additional file 1: Figure S1.** Intracellular accumulation of the HyHEL-Fab in control, Δ*atg8* and Δ*atg11* strains. Intracellular levels of HyHEL-Fab were detected by Western blot of cell lysates. Equal amounts of total intracellular protein (determined by BCA assay after cell lysis) were loaded on the gel. **Figure S2.** Full size microscopic images of vacuolar membrane staining of Fab#34, ∆*vam6*, ∆*vps41,* ∆*ypt7* with FM4-64 shown in Fig. 2. **Figure S3.** Vacuolar membrane staining of HyHEL Fab and CES Δ*ypt7* and additional protease disruptions (Δ*pep4*, Δ*prb1*). **Figure S4.** Agarose gels of PCRs confirming gene disruptions in genomic DNA of *P. pastoris*. **Figure S5.** Growth kinetics of *P. pastoris* in 2 mL screening cultures based on glucose-release rates of 12 mm glucose Feed Beads.

**Additional file 2: Table S1.** Fold changes of titer, wet cell weight and product yield (titer per wet cell weight) of engineered *P. pastoris* strains in comparison to Fab#34 or CES#18, respectively obtained in small scale screenings. **Table S2.** Fold changes of volumetric productivity Q_P_, specific productivity q_P_, biomass yield Y_X/S_ and specific growth rate µ of engineered *P. pastoris* strains in comparison to Fab#34 obtained in fed batch bioreactor cultivations at the end of cultivation. **Table S3.** Primer sequences used for generation of the split marker cassettes and detection of positive transformants.

## Data Availability

All data generated or analysed during this study are included in this published article and its additional files.
