# Supplementary material for: Disruption of vacuolar protein sorting components of the HOPS complex leads to enhanced secretion of recombinant proteins in Pichia pastoris
Source: Microb Cell Fact. 2019 Jul 3;18:119. doi: 10.1186/s12934-019-1155-4 (PMC6607557; doi:10.1186/s12934-019-1155-4)

Additional File 1 to Marsalek et al.

Disruption of HOPS components reduces vacuolar protein sorting and enhances recombinant protein secretion in *Pichia pastoris*

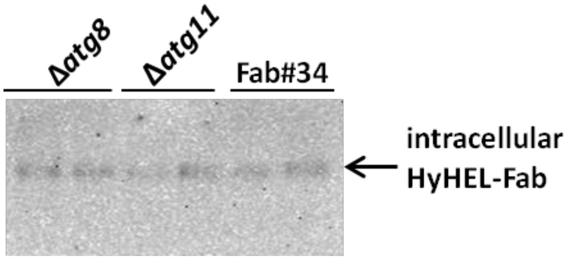

Additional Figure S1: Intracellular accumulation of the HyHEL-Fab in control, Δ*atg8* and Δ*atg11* strains. Intracellular levels of HyHEL-Fab were detected by Western blot of cell lysates. Equal amounts of total intracellular protein (determined by BCA assay after cell lysis) were loaded on the gel.

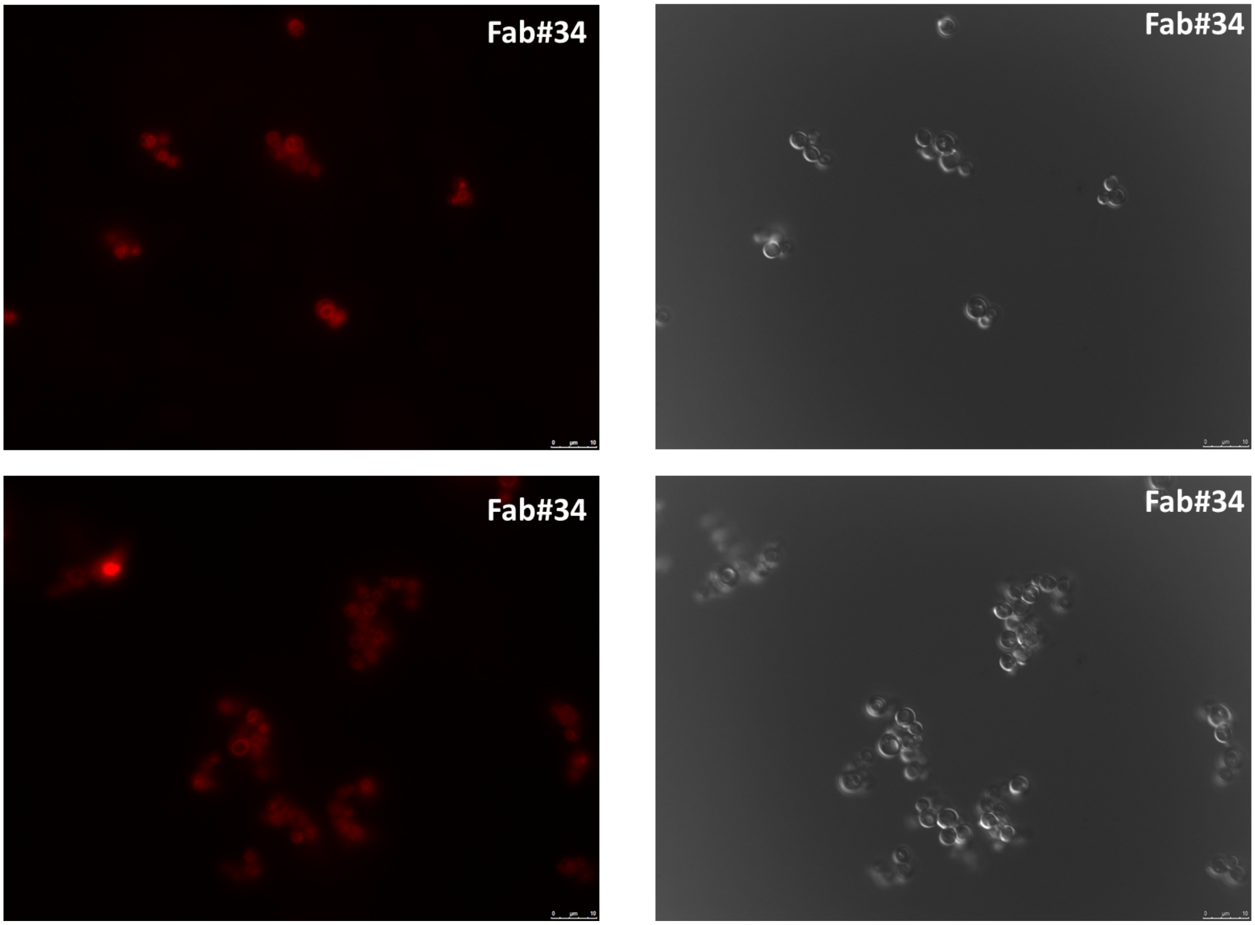

Additional Figure S2a: Full size microscopic images of vacuolar membrane staining of Fab#34 shown in Figure 2. The vacuolar membrane was stained with FM4-64 and the cells were viewed in an epifluorescence microscope. The fluorescence and the DIC images of two representative images per construct are shown. Bar, 10 µm.

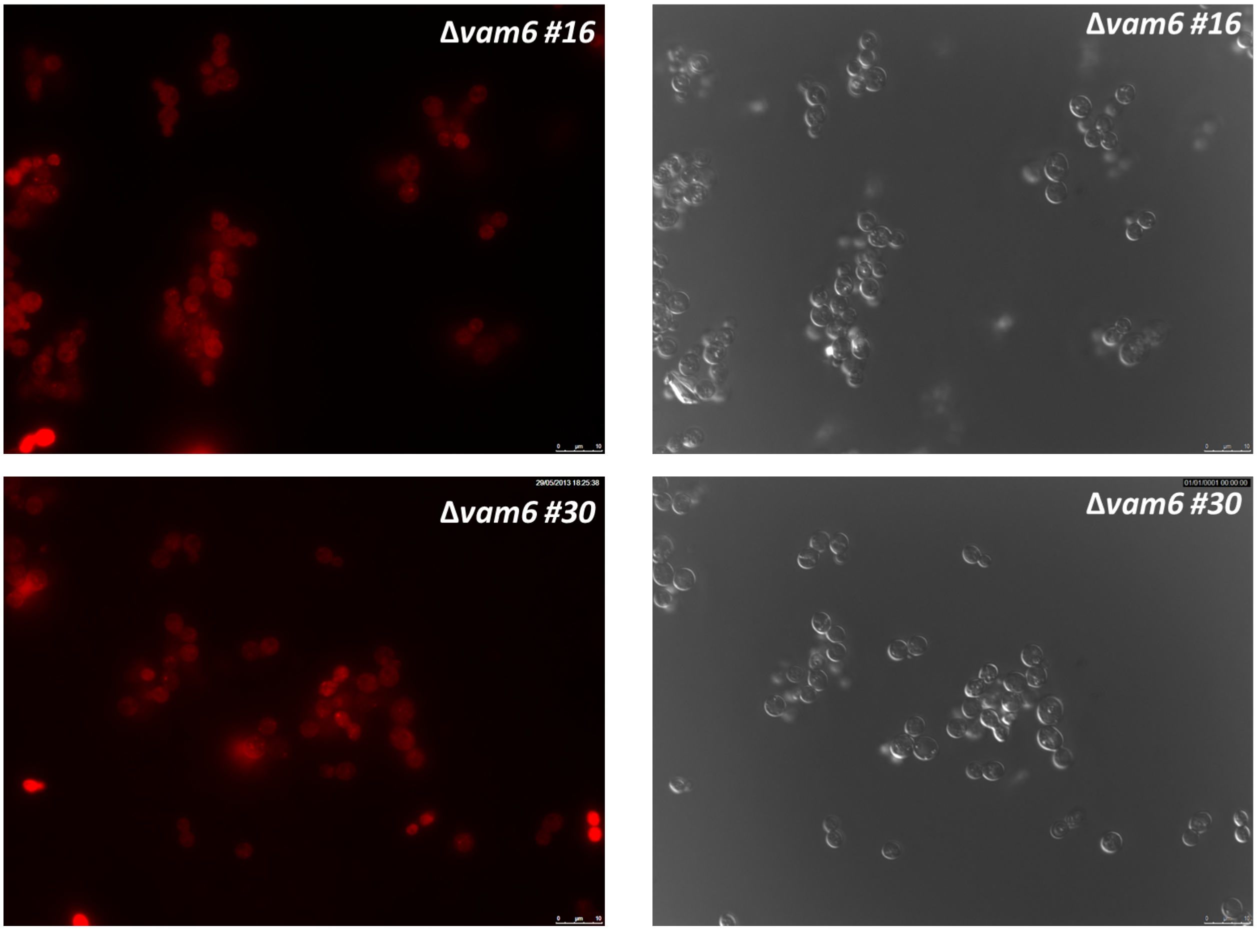

Additional Figure S2b: Full size microscopic images of vacuolar membrane staining of Fab#34 Δ*vam6* shown in Figure 2. The vacuolar membrane was stained with FM4-64 and the cells were viewed in an epifluorescence microscope. The fluorescence and the DIC images of two representative images per construct are shown. Bar, 10 µm.

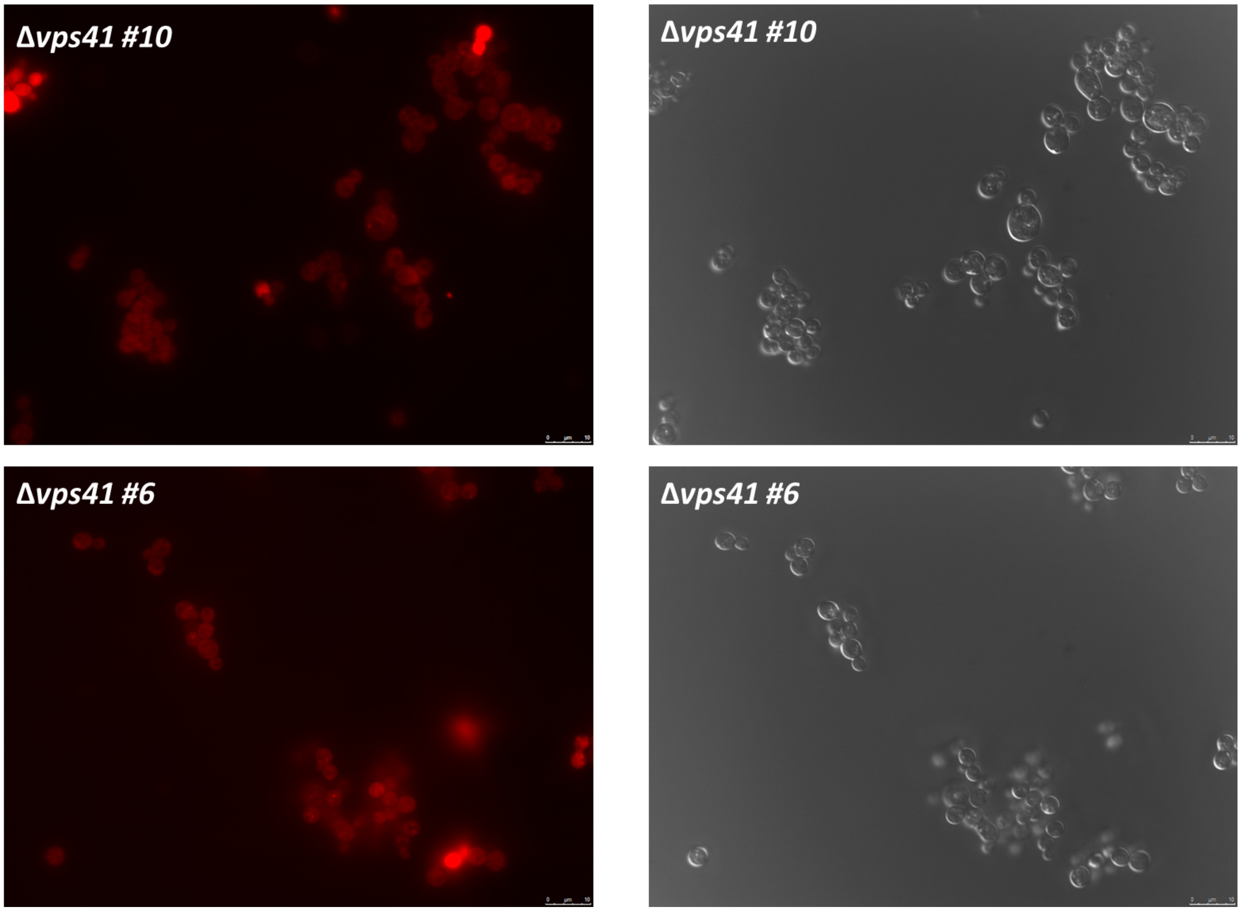

Additional Figure S2c: Full size microscopic images of vacuolar membrane staining of Fab#34 Δ*vps41* shown in Figure 2. The vacuolar membrane was stained with FM4-64 and the cells were viewed in an epifluorescence microscope. The fluorescence and the DIC images of two representative images per construct are shown. Bar, 10 µm.

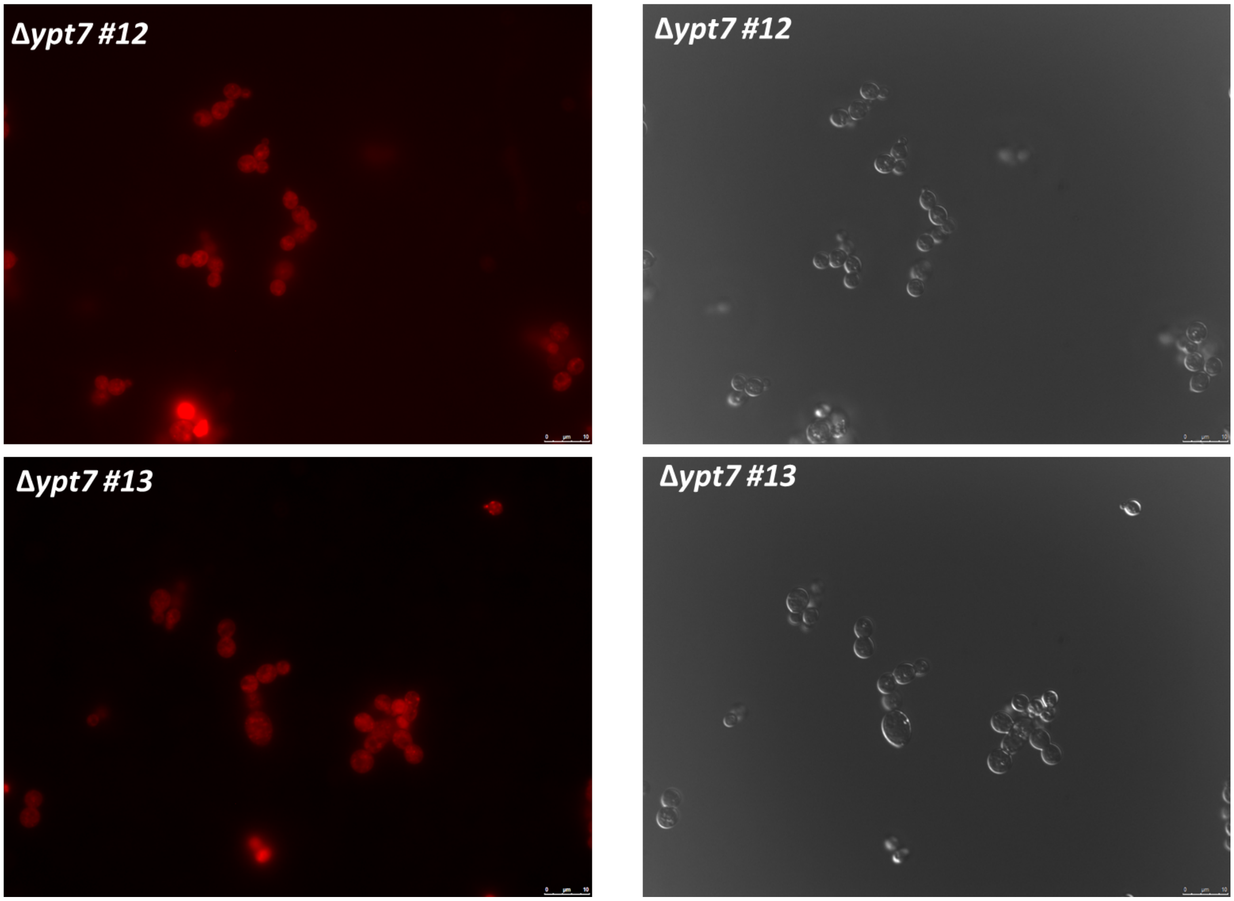

Additional Figure S2d: Full size microscopic images of vacuolar membrane staining of Fab#34 Δ*ypt7* shown in Figure 2. The vacuolar membrane was stained with FM4-64 and the cells were viewed in an epifluorescence microscope. The fluorescence and the DIC images of two representative images per construct are shown. Bar, 10 µm.

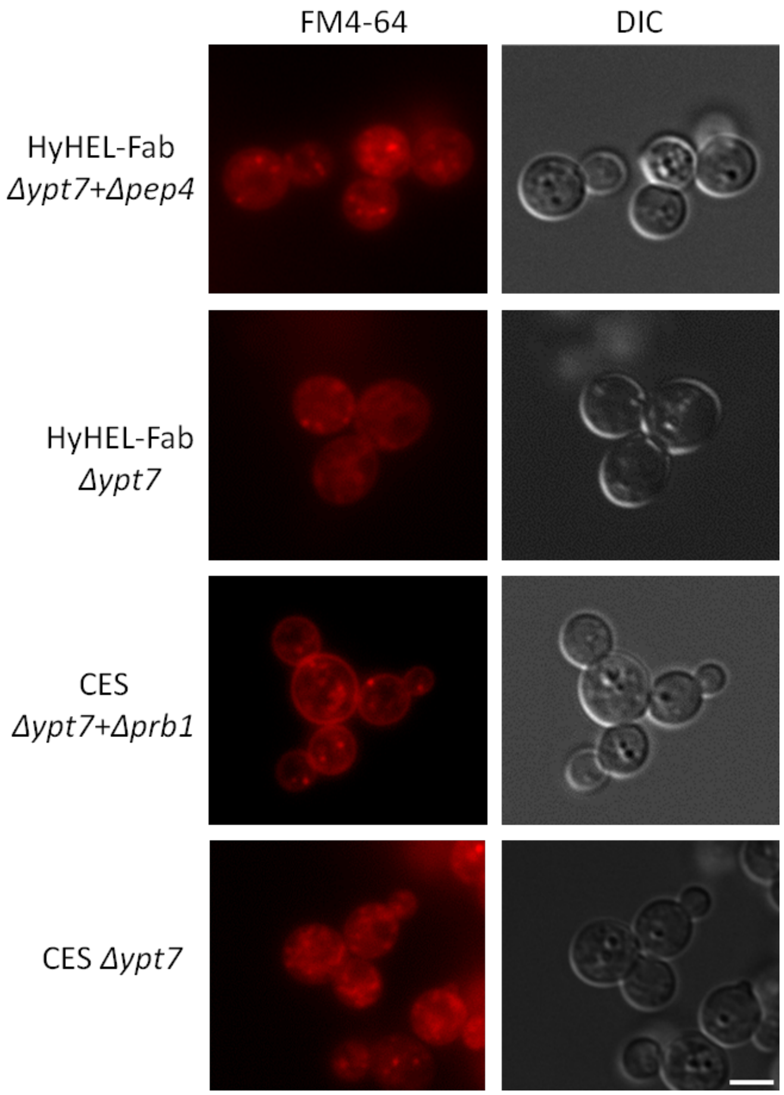

Additional Figure S3: Vacuolar membrane staining of HyHEL-Fab and CES Δ*ypt7* and additional protease disruptions (Δ*pep4*, Δ*prb1*). The vacuolar membrane was stained with FM4-64 and the cells were viewed in an epifluorescence microscope. The fluorescence and the DIC images are shown. Bar, 3 µm.

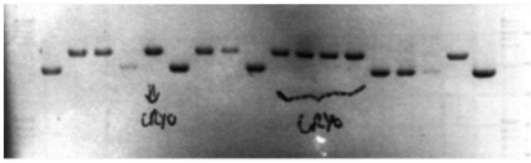

CES#18 Δ*vam6*

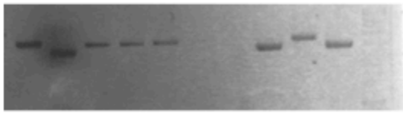

CES#18 Δ*vps41*

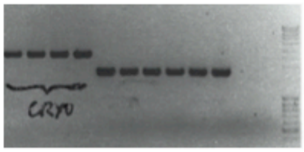

CES#18 Δ*ypt7*

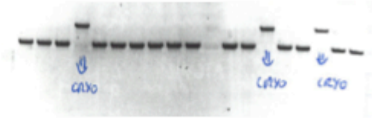

CES#18 Δ*atg8*

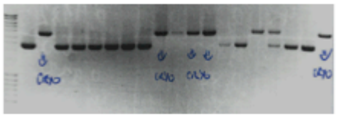

CES#18 Δ*atg11*

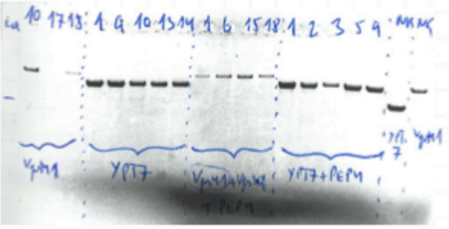

Fab#34 Δ*vps41* / Δ*ypt7* / Δ*vps41* / Δ*ypt7*

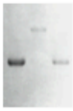

Fab#34 Δ*vam6*

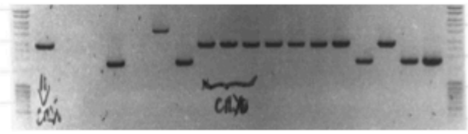

Fab#34 Δ*ypt7*

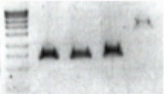

Fab#34 Δ*atg11*

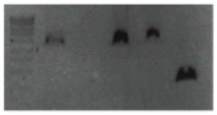

Fab#34 Δ*atg8*

mutation	bp wild type	bp mutant
Δ*vps41*	2995	4065
Δ*ypt7*	1771	3244
Δ*vam6*	2248	3617
Δ*prb1*	2296	3744
Δ*atg8*	1549	3150
Δ*atg11*	2386	3646

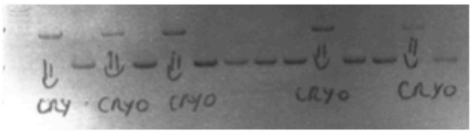

CES#18 Δ*ypt7*Δ*pep4*

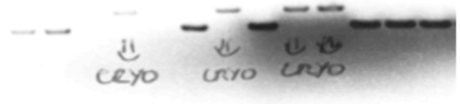

CES#18 Δ*ypt7*Δ*vps70*

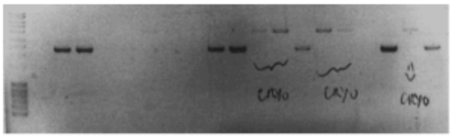

CES#18 Δ*ypt7*Δ*prb1*

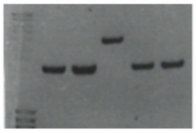

CES#18 Δ*vam6*Δ*pep4*

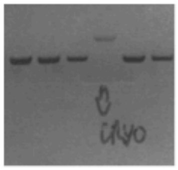

CES#18 Δ*vam6*Δ*vps70*

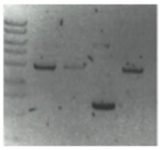

CES#18 Δ*vam6*Δ*prb1*

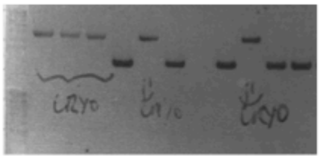

Fab#34 Δ*ypt7*Δ*pep4*

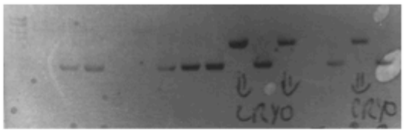

Fab#34 Δ*ypt7*Δ*vps70*

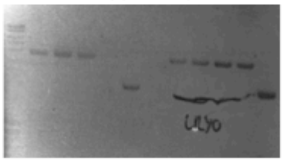

Fab#34 Δ*vam6*Δ*pep4*

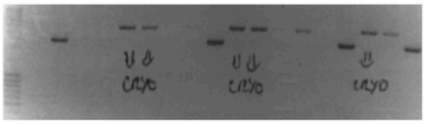

Fab#34 Δ*vam6*Δ*vps70*

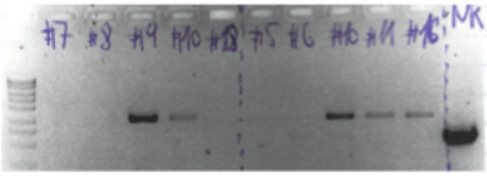

Fab#34
Δvam6Δpep4 / Δvam6Δvps70
confirming VAM6

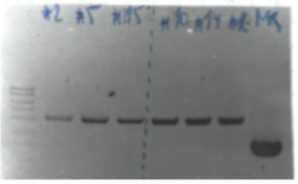

Fab#34 / CES#18
Δypt7Δvps70
confirming VPS70

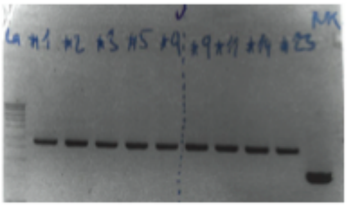

Fab#34
Δypt7Δpep4 / Δypt7Δvps70
confirming YPT7

Additional Figure S4: Agarose gels of PCRs confirming gene disruptions in genomic DNA of *P. pastoris*.

Additional Figure S5: Growth kinetics of *P. pastoris* in 2 mL screening cultures based on glucose-release rates of 12 mm glucose Feed Beads releasing glucose at a rate of 1.63 $t^{0.74}$ mg per disc. Cells were inoculated with an OD_{600} of 1.0 and cultivated in M2 medium with one glucose feed bead for 48 h with shaking at 250 rpm and 25°C.

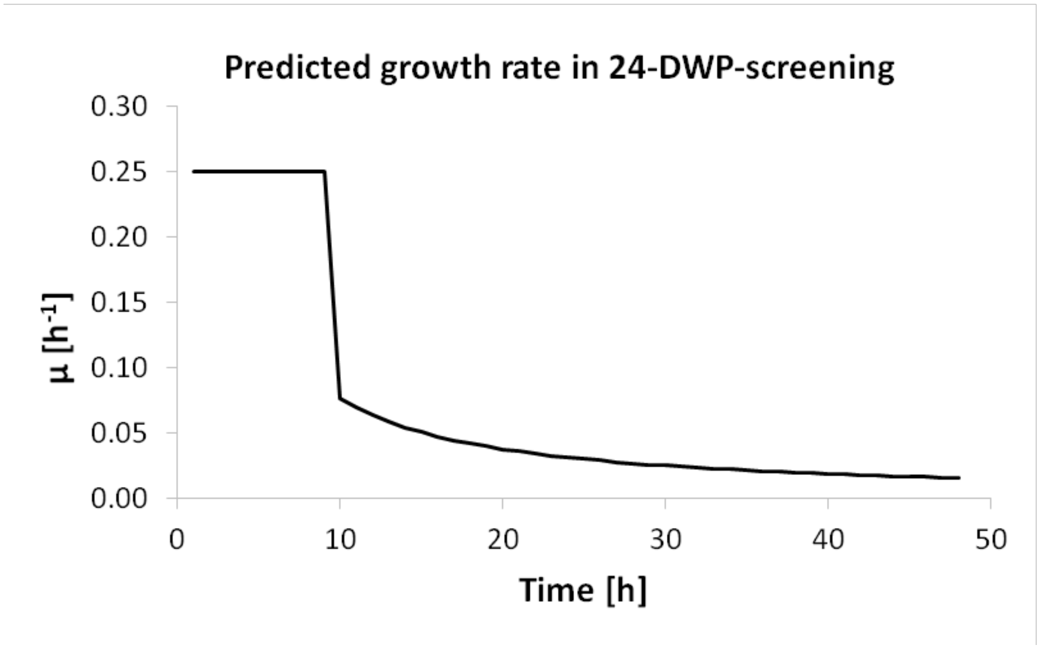

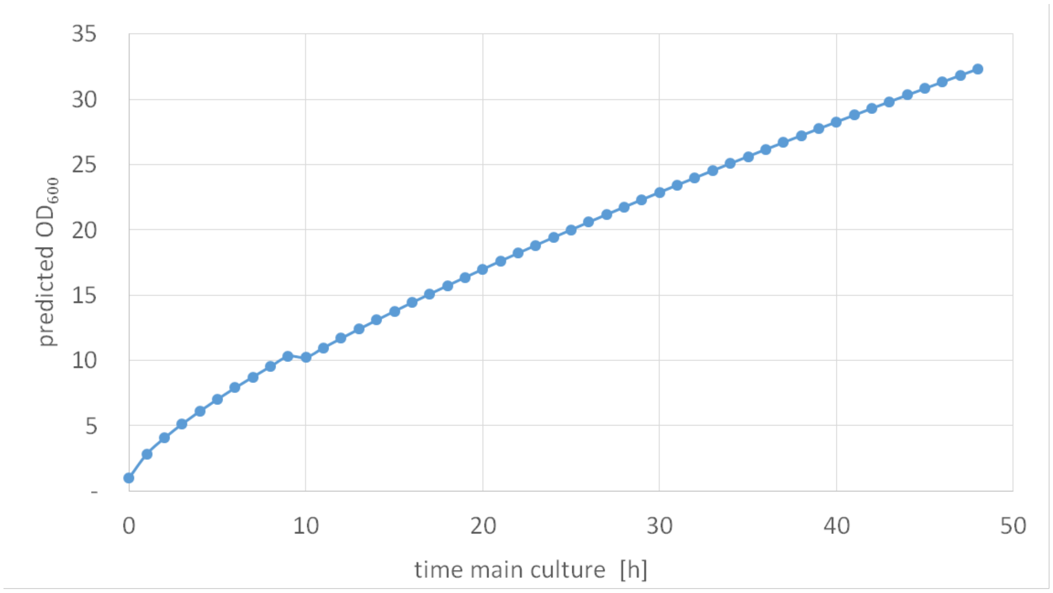

Supplement: Supplementary file 1 — Additional file 1: Figure S1. Intracellular accumulation of the HyHEL-Fab in control, Δatg8 and Δatg11 strains. Intracellular levels of HyHEL-Fab were detected by Western blot of cell lysates. Equal amounts of total intracellular protein (determined by BCA assay after cell lysis) were loaded on the gel. Figure S2. Full size microscopic images of vacuolar membrane staining of Fab#34, ∆vam6, ∆vps41, ∆ypt7 with FM4-64 shown in Fig. 2. Figure S3. Vacuolar membrane staining of HyHEL Fab and CES Δypt7 and additional protease disruptions (Δpep4, Δprb1). Figure S4. Agarose gels of PCRs confirming gene disruptions in genomic DNA of P. pastoris. Figure S5. Growth kinetics of P. pastoris in 2 mL screening cultures based on glucose-release rates of 12 mm glucose Feed Beads. [file 12934_2019_1155_MOESM1_ESM.pdf]
