# Supplementary material for: Disruption of vacuolar protein sorting components of the HOPS complex leads to enhanced secretion of recombinant proteins in Pichia pastoris
Source: Microb Cell Fact. 2019 Jul 3;18:119. doi: 10.1186/s12934-019-1155-4 (PMC6607557; doi:10.1186/s12934-019-1155-4)
Supplement: Supplementary file 2 — Additional file 2: Table S1. Fold changes of titer, wet cell weight and product yield (titer per wet cell weight) of engineered P. pastoris strains in comparison to Fab#34 or CES#18, respectively obtained in small scale screenings. Table S2. Fold changes of volumetric productivity QP, specific productivity qP, biomass yield YX/S and specific growth rate µ of engineered P. pastoris strains in comparison to Fab#34 obtained in fed batch bioreactor cultivations at the end of cultivation. Table S3. Primer sequences used for generation of the split marker cassettes and detection of positive transformants. [file 12934_2019_1155_MOESM2_ESM.pdf]

Additional File 2 to Marsalek et al.

Disruption of HOPS components reduces vacuolar protein sorting and enhances recombinant protein secretion in *Pichia pastoris*

Additional Table S1: Fold changes (FC) of titer, wet cell weight (WCW) and product yield (titer per wet cell weight) of engineered *P. pastoris* strains in comparison to Fab#34 or CES#18, respectively obtained in small scale screenings.

After 48 h of cultivation in M2+feed bead, Fab#34 reached the following: Fab titer of 0.71 ± 0.08 mg/L, biomass concentration of 42.6 ± 1.69 g/L WCW and Fab yield of 0.017 ± 0.001 mg Fab/g WCW.

After 48 h of cultivation in BM+feed bead, CES#18 reached the following: CES titer of 50.55 ± 5.13 mg/L, biomass concentration of 55.27 ± 2.18 g/L WCW and CES yield of 0.890 ± 0.070 mg CES/g WCW.

Genotype	Strain name	FC titer	FC yield	FC WCW	FC titer	FC yield	FC WCW	
atg8::HphMX	*Δatg8*	0.56 ± 0.13	0.71 ± 0.16	0.75 ± 0.00	1.16 ± 0.09	1.18 ± 0.09	0.98 ± 0.01	this study
atg11::HphMX	*Δatg11*	0.51 ± 0.15	0.58 ± 0.15	0.81 ± 0.04	1.13 ± 0.06	1.14 ± 0.07	0.99 ± 0.01	this study
vam6::KanMX	*Δvam6*	1.23 ± 0.12	1.41 ± 0.13 *	0.90 ± 0.03 *	1.40 ± 0.12 **	1.62 ± 0.08 ***	0.94 ± 0.07	this study
ypt7::KanMX	*Δypt7*	1.26 ± 0.06 **	1.46 ± 0.08 ***	0.89 ± 0.04 *	1.64 ± 0.16 **	1.43 ± 0.11 **	1.10 ± 0.08	this study
vps41::KanMX	*Δvps41*	0.96 ± 0.09	1.02 ± 0.11	0.96 ± 0.03	1.07 ± 0.05	1.21 ± 0.06	0.89 ± 0.02 **	this study
prb1::HphMX	*Δprb1*	< 0.5 (WB)	< 0.5 (WB)	1.00 ± 0.07	0.78 ± 0.15 *	0.84 ± 0.18	0.95 ± 0.04	Marsalek et al. 2017
Δvam6 prb1::HphMX*	*Δvam6Δprb1*	-	-	-	1.01 ± 0.04	1.18 ± 0.04 *	0.85 ± 0.01 ***	this study
Δvam6::KanMX / Δvam6 pep4::HphMX*	*Δvam6Δpep4*	1.52 ± 0.07 ***	1.78 ± 0.10 ***	0.87 ± 0.02 ***	1.47 ± 0.03 **	1.75 ± 0.03 ***	0.84 ± 0.00 ***	this study
vam6::KanMX vps70::HphMX	*Δvam6Δvps70*	1.47 ± 0.14 **	1.53 ± 0.11 ***	0.95 ± 0.03	1.35	1.35	1.00	this study
Δypt7 prb1::HphMX*	*Δypt7Δprb1*	-	-	-	1.15 ± 0.08	1.36 ± 0.10 *	0.84 ± 0.03 **	this study
ypt7::KanMX pep4::HphMX	*Δypt7Δpep4*	1.41 ± 0.10 **	1.65 ± 0.13 ***	0.87 ± 0.02 ***	1.57 ± 0.08 **	1.83 ± 0.08 ***	0.85 ± 0.01 ***	this study
ypt7::KanMX vps70::HphMX	*Δypt7Δvps70*	1.24 ± 0.10	1.49 ± 0.13 **	0.85 ± 0.02 ***	1.07 ± 0.06	1.25 ± 0.08	0.85 ± 0.02 ***	this study
Δvam6 vps41::KanMX*	*Δvam6Δvps41*	1.11	1.29	0.86	-	-	-	this study
Δypt7 vam6::KanMX*	*Δypt7Δvam6*	0.81 ± 0.15	1.14 ± 0.25	0.82 ± 0.03	-	-	-	this study
vps8::KanMX	*Δvps8*	0.30 ± 0.12 ***	0.43 ± 0.15 **	0.94 ± 0.02 *	0.56 ± 0.05 ***	0.73 ± 0.07 ***	0.81 ± 0.04 ***	Marsalek et al. 2017
vps21::KanMX	*Δvps21*	0.50 ± 0.11 **	0.51 ± 0.11 **	0.96 ± 0.02	0.76 ± 0.03 ***	0.87 ± 0.04 **	0.86 ± 0.02 ***	Marsalek et al. 2017
vps8::KanMX prb1::HphMX	*Δvps8Δprb1*	0.00	0.00	0.92	1.15 ± 0.12	1.57 ± 0.17 ***	0.78 ± 0.04 **	Marsalek et al. 2017
vps21::KanMX prb1::HphMX	*Δvps21Δprb1*	0.10 ± 0.09 ***	0.12 ± 0.10 ***	0.79 ± 0.03 ***	1.51 ± 0.09 ***	1.67 ± 0.11 ***	0.83 ± 0.02 ***	Marsalek et al. 2017

Δvam6 vps8::KanMX*	*Δvam6Δvps8*	not viable	not viable	not viable	0.09 ± 0.04 ***	0.12 ± 0.05 ***	0.90 ± 0.08	this study
Δvam6 vps8::KanMX prb1::HphMX*	*Δvam6Δvps8Δprb1*	not viable	not viable	not viable	1.15 ± 0.09	1.66 ± 0.17 **	0.62 ± 0.08	this study
Δvam6 Δvps41* prb1::HphMX*	*Δvam6Δvps41Δprb1*	0.45 ± 0.04 ***	0.56 ± 0.04 ***	0.80 ± 0.03 **	-	-	-	this study
Δvam6 vps41::KanMC pep4::HphMX*	*Δvam6Δvps41Δpep4*	0.66 ± 0.11	0.87 ± 0.14	0.85 ± 0.03	-	-	-	this study
Δvam6 Δypt7* prb1::HphMX*	*Δvam6Δypt7Δprb1*	0.16 ± 0.03 ***	0.21 ± 0.03 ***	0.74 ± 0.04 **	-	-	-	this study
Δypt7 vam6::KanMX pep4::HphMX*	*Δvam6Δypt7Δpep4*	0.95 ± 0.05	1.23 ± 0.09	0.88 ± 0.02	-	-	-	this study
pPM2aK20-P_{GAP}*-KAR2*	*KAR2OE*	1.11 ± 0.13	1.05 ± 0.10	0.95 ± 0.03	-	-	-	this study
pPM2aK20-P_{GAP}*-SBH1*	*SBH1OE*	2.41 ± 0.22 ***	2.25 ± 0.19 ***	0.98 ± 0.03	-	-	-	this study
pPM2aK20-P_{GAP}*-RHO4*	*RHO4OE*	1.31 ± 0.09 *	1.11 ± 0.07	1.09 ± 0.03 **	-	-	-	this study
pPM2aK20-P_{GAP}*-SBH1*	*SBH1OE*	2.64 ± 0.56	2.78 ± 0.6	0.95 ± 0.02	-	-	-	this study
Δypt7 + pPM2aK20-*P_{GAP}*-SBH1*	*Δypt7+SBH1OE*	3.71 ± 0.35 **	3.96 ± 0.33 **	0.93 ± 0.03	-	-	-	this study
Δvam6 pep4::HphMX + pPM2aK20-*P_{GAP}*-SBH1*	*Δvam6Δpep4+SBH1OE*	2.77 ± 0.28 ***	2.86 ± 0.25 ***	0.96 ± 0.03	-	-	-	this study

[a] *Marker recycled with Cre recombinase; loxP site left at KO locus. [b] Fold changes compared to the parent ± standard error of the mean are given. Statistical significance was determined by the Student's t-test and indicated with an asterisk ($p < 0.05$ = *, $p < 0.01$ = **, $p < 0.001$ = ***).

Additional Table S2: Fold changes (FC) of volumetric productivity Q_P, specific productivity q_P, biomass yield $Y_{X/S}$ and specific growth rate μ of engineered *P. pastoris* strains in comparison to Fab#34 obtained in fed batch bioreactor cultivations at the end of cultivation (FC of data presented in Figure 8 and Table 1).

	**FC mean Q_p**	**FC mean q_p**	**FC $Y_{X/S}$**	**FC mean μ**
Δvam6Δpep4	1.68	1.74	1.06	0.95
**Δ*vam6*Δpep4+*SBH1*OE**	1.98 ± 0.31	1.90 ± 0.40	1.04 ± 0.04	1.16 ± 0.16
**Δ*ypt7***	1.58 ± 0.14	1.75 ± 0.18	0.97 ± 0.14	1.05 ± 0.06
***SBH1*OE**	1.68	1.44	0.83	0.95
**Δ*ypt7+SBH1*OE**	2.48 ± 0.00	1.79 ± 0.14	1.06 ± 0.03	1.02 ± 0.10

Additional Table S3: Primer sequences used for generation of the split marker cassettes and detection of positive transformants

Gene name	ORF ID	Primer A fw	Primer A bw	Primer D fw	Primer D bw	DET primer fw	DET primer bw
ATG8	PP7435_ Chr4-0258	CAATGGCCGATAC AAACTCTTC	GTTGTCGACCTGCAGCGTACTAG CCTTTTCACTCATCGTC	TAGGTGATATCAGATCCACTGCA ATTTAAAGACGAACACCC	CAAAAAACAGTAA CAAAGCCAGCC	TATTAATAACTGCA AGACCGGC	ACAATGCATGAGA ACGAGTG
ATG11	PP7435_ Chr1-1358	AGTAGAATTGGGC TGCAAAG	GTTGTCGACCTGCAGCGTACCGA TGGTACTCAGAAGGGGG	TAGGTGATATCAGATCCACTATC AAGCCAATGAGAAGTCC	CTTTAGATTACCAG CGAGCC	CTGAAATAATAGG CAAACGGGAC	TGAACCCGTCAAT CCATTTG
VAM6	PP7435_ Chr1-0295	TTTTCTGGTCCCTT GATCCTCGC	GTTGTCGACCTGCAGCGTACCGG GATTTACGTATACTAGAGCAG	TAGGTGATATCAGATCCACTATG TGTTCTCAATTGGCGATCTTAC	CCTGAAGTTGGCT GTCCGGAG	GGTCATAAGGAGA TCGCGAATAAC	TATGGCTGTTTGC AATGAATGTTCG
VPS41	PP7435_ Chr4-0955	TTCTATCCGTTT CATGCTTCAAGT G	GTTGTCGACCTGCAGCGTACTT CGCTGATCCTTAACTTGCCTAG	TAGGTGATATCAGATCCACTT CAAACGTCCTGTGCATGCTGTG	GAGTTCGGGAAA TACACCTTGGG	GTTCGTTAGTGG CCACAATAGTAG	TTTTCCAGTCAG TTAGATAATGTT CC
YPT7	PP7435_ Chr4-0052	TTCACAAGAGCAG ATAAAGGAGATG	GTTGTCGACCTGCAGCGTACCCT TGGTGATTTTGCCGGTCTTG	TAGGTGATATCAGATCCACTGAG GAGCAGATTGTTGTGTACTAG	TAAGCGAAACGAG GCATACACCC	ACATAACAAGACA ACTCGTACAACC	GTAAAGATAACCA AGAAAACAAGAGC
PEP4	PP7435_ Chr3-0072	TCACTGCCAGCAT CTTTTACTCAC	GTTGTCGACCTGCAGCGTACTTC TAAATAGTTGCCACCTTATCGG	TAGGTGATATCAGATCCACTTGG TTTTGCCGTTGAAGCTTCGC	CGGCGAAGGTTAA AGTTAAGTCTG	TCGCTCCCCGTAC GTTAAGAATG	CTAATACATGACC CAGAAACCTCC
PRB1	PP7435_ Chr1-0540	GCCTACATCAATA ATACCAATCATCG	GTTGTCGACCTGCAGCGTACGTA ACTTGAAACATGAGGCGTGAC	TAGGTGATATCAGATCCACTGAA GAAGATGAAAGAAGGCCACCC	AAGCAGGAGATTT ACCACCACCC	TACATCTACTTTAA CATCAATCATCTC	GCTGCTGGCGAGG TGTTACAAG
VPS70	PP7435_ Chr3-0372	CGTCACCTAAGCA AGAGCCTAAG	GTTGTCGACCTGCAGCGTACACT CAACTAGATAGAATGGCAATGG	TAGGTGATATCAGATCCACTACG GATTGGTTGAGTTTACTAAGTC	GAACAAAGATGGC GAGGCACCC	AGCTCAAGATACA TTCACCCCAG	AGTCTCGGTTGAA TTCTTGAAAGTG
